# Material properties and structure of natural graphite sheet

**DOI:** 10.1038/s41598-020-75393-y

**Published:** 2020-10-29

**Authors:** Martin Cermak, Nicolas Perez, Michael Collins, Majid Bahrami

**Affiliations:** 1 Laboratory for Alternative Energy Conversion, School of Mechatronic Systems Engineering, Faculty of Applied Science, Simon Fraser University, 250-13450 102 Avenue, Surrey, BC V3T 0A3 Canada; 2 The Institut National des Sciences Appliquées de Lyon, 20 Avenue Albert Einstein, 69621 Villeurbanne Cedex, France; 3 Solar Thermal Research Laboratory, Department of Mechanical and Mechatronics Engineering, University of Waterloo, 200 University Avenue West, Waterloo, ON N2L 3G1 Canada

**Keywords:** Energy science and technology, Engineering, Materials science

## Abstract

Natural graphite sheet (NGS) is compressible, porous, electrically and thermally conductive material that shows a potential to be used in fuel cells, flow batteries, electronics cooling systems, supercapacitors, adsorption air conditioning, and heat exchangers. We report the results of an extensive material characterization study that focuses on thermal conductivity, thermal diffusivity, electrical conductivity, coefficient of thermal expansion (CTE), compression strain, and emissivity. All the properties are density-dependent and highly anisotropic. Increasing the compression from 100 to 1080 kPa causes the through-plane thermal and electrical conductivities to increase by up to 116% and 263%, respectively. The properties are independent of the sheet thickness. Thermal and electrical contact resistance between stacked NGS is negligible at pressures 100 to 1080 kPa. In the in-plane direction, NGS follows the Wiedemann-Franz law with Lorenz number 6.6 $\times$ 10$^{-6}$ W $\Omega$ K$^{-2}$. The in-plane CTE is low and negative (shrinkage with increasing temperature), while the through-plane CTE is high, increases with density, and reaches 33 $\times$ 10$^{-6}$ K$^{-1}$. Microscope images are used to study the structure and relate it to material properties. An easy-to-use graphical summary of the forming process and NGS properties are provided in Appendices A and B.

## Introduction

Natural graphite sheet (NGS) is a compressible, porous, electrically and thermally conductive material that has been used primarily to make sealing gaskets because of its ability to conform to rough surfaces, withstand high temperatures, and resist corrosive fluids. Presently, it is used—or considered to be used—in numerous applications, including bipolar plates in fuel cells and flow batteries for its electrical conductivity and chemical stability^1,2,3^, supercapacitors for its corrosion resistance^4,5^, adsorption cooling systems for its high thermal diffusivity^6^, heat spreaders for its high in-plane thermal conductivity^7,8^, heat exchangers for its thermal conductivity and corrosion resistance^9^, and heat sinks for its thermal conductivity, low weight, and ease of forming into complex shapes^10,11^. Multiple other names are used in the literature to refer to NGS, such as compressed exfoliated natural graphite (CENG), graphite foil, or flexible graphite. However, the term flexible graphite can be misleading because flexing thicker sheets results in a brittle fracture.

NGS is manufactured from natural graphite flakes in a process that involves soaking the flakes in an intercalation compound that penetrates between the graphite layers. During subsequent rapid heating, the vaporized compound forces the graphite layers apart, forming exfoliated natural graphite (ENG) particles also known as worms^13^. NGS at densities 0.5 to $1.73\,\hbox {g\, cm}^{-3}$ is formed by compressing ENG particles at pressures ranging from 1  to 31 MPa^6,14,15^.

In this study, we measure an extensive set of material properties and relate them to the structure observed by an SEM microscope. The sheet density is the primary parameter with the strongest influence on the properties. We found the through-plane thermal and electrical conductivity to increase with through–plane compression, and observed that the increase is density-dependent. The thickness of the sheet did not affect the measured properties.

The through–plane conductivities are low (${2}\,\hbox {Wm}^{-1}\hbox {K}^{-1}$ to ${6}\,\hbox {Wm}^{-1}\hbox {K}^{-1}$ and ${3}\,\hbox {Scm}^{-1}$ to ${25}\,\hbox {Scm}^{-1}$) while the in-plane ones are high (${100}\,\hbox {Wm}^{-1}\hbox {K}^{-1}$ to ${350}\,\hbox {Wm}^{-1}\hbox {K}^{-1}$ and ${500}\,\hbox {Scm}^{-1}$ to ${1700}\,\hbox {Scm}^{-1}$). In the in–plane direction, both thermal and electrical conductivities increase linearly with density. At low densities, our results agree with the previous studies, but at high densities we observed values 20 % to 25 % lower than those reported in the literature^17,18,20^. In the through–plane direction, the conductivities decrease with density. The data from previous studies^6,19,20,21,22^ show high scatter and incomplete coverage of the density spectrum, which prevented us from forming a clear comparison with our results. The thermal and electrical contact resistance between stacked sheets of NGS is negligible at pressures higher than 100 kPa. We found the emissivity at wavelengths from $2\,$ to $26\,\upmu \hbox {m}$ to decrease with density in the range 0.39 to 0.52.

When heated from room temperature to $100^{\circ }C$, NGS shows a dimensional stability superior to conventional materials such as aluminum or copper, however, the expansion in the through-plane direction is high and strongly dependent on the density. Our measurements show a very low and negative coefficient of thermal expansion (CTE) in the in–plane direction ($-0.6\times 10^{-6}{\hbox {K}^{-1}}$ to $-1.4\times 10^{-6}{\hbox {K}^{-1}}$) while in the through-plane direction it is positive and ranges from $10\times 10^{-6}\,{\hbox {K}^{-1}}$ to $33\times 10^{-6}\,\hbox {K}^{-1}$. Coupling the results with our study of the compression behaviour (outlined here and detailed elswhere^15^) allows for a quantitative analysis of thermomechanical stresses in devices such as fuel cells.

The structure of NGS is composed of tens-of-nanometers thin, highly anisotropic graphitic features whose orientation, spacing, and contact quality dictate the overall macroscopic properties of NGS. Compressing NGS causes reversible and irreversible structural changes that reflect in the overall properties. The irreversible changes happen during the forming process, and the reversible ones during the low-pressure loading of a finished sheet^15^.

In summary, the goal of the present study is to compile a comprehensive resource describing the NGS properties and their relationship with the material structure. While the authors believe that this is the most comprehensive resource to date, we clearly state the need for further research. The most pressing issue is the lack of understanding of how the parameters of exfoliation methods and raw graphite flakes affect the structure and material properties of NGS.

## Results

We first describe the structure of NGS, and then address each of the measured material properties. The forming process is not covered in this publication, however, a brief summary is provided in Appendix A. The reader is referred to other publication^15^ for details. Alongside the detailed description of the measurements and results further in this section, a simplified graphical summary of the measured material properties with explicit formulas of best fits is provided in Appendix B.

### Structure

The structure of NGS is a result of compaction of ENG particles that are composed of highly crystalline cell walls^13^ as can be seen in Appendix A. The cell walls are 15 nm to 60 nm thick^14,37^ and their dimensions in the *a* and *b* crystal directions are equal to or less than the size of raw flakes^38^. Upon compression, the cell walls within a single ENG particle deform into a platelet shape^32^ but the particles remain cohesive^14^.

**Figure 1:**
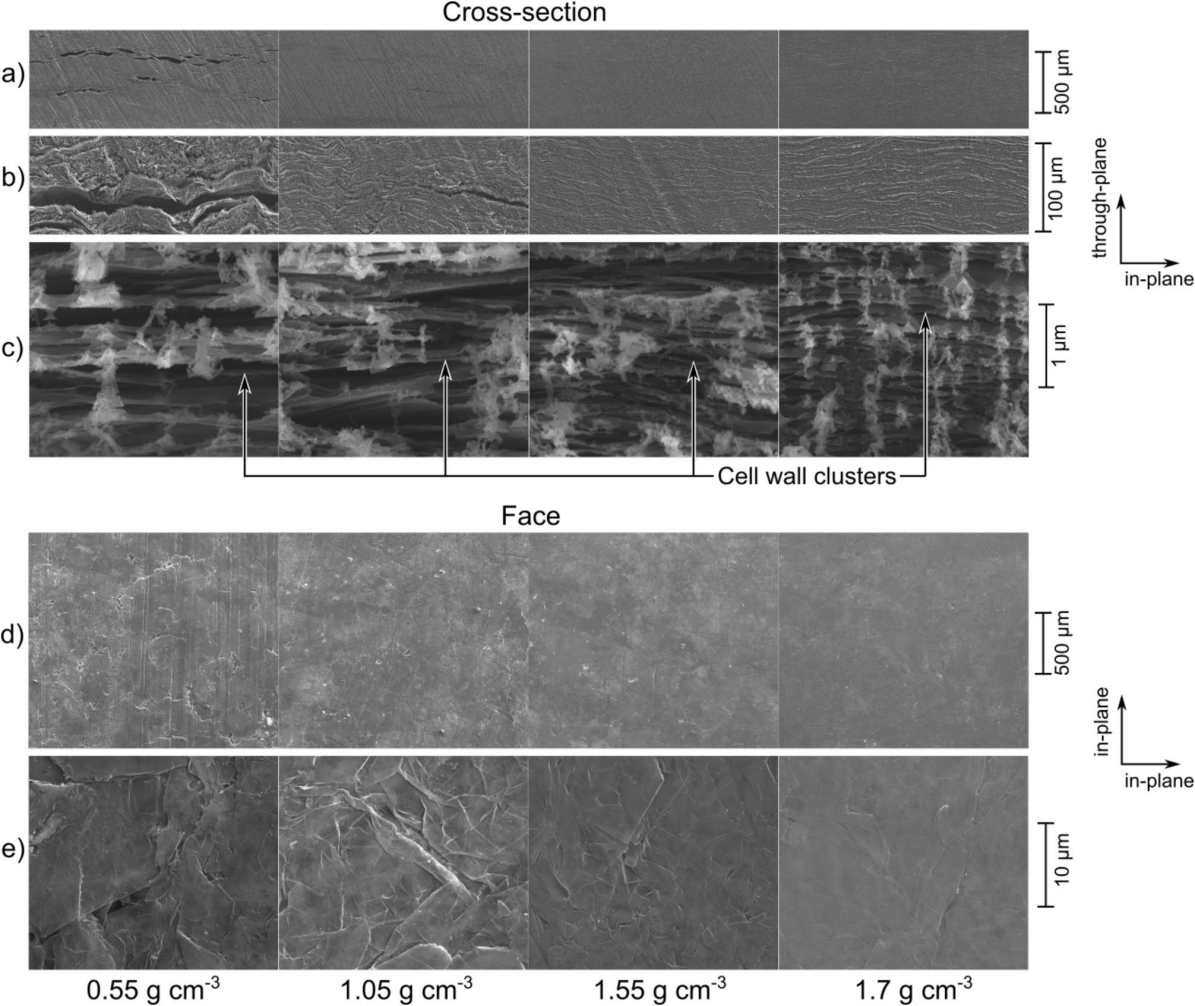
Microscope images of NGS cross-section **(a–c)** and face **(d, e)**. The high-magnification cross-section images c) have been rotated to demonstrate the change of thickness and spacing of cell walls and they do not represent the orientation angle accurately. High-resolution images are available in the supplementary dataset^36^.

The cross-section structure at macro scale (hundreds of micrometers, Fig. 1a) changes significantly with the sheet density. At low densities, it is heterogenous with large slit shape pores, and becomes homogeneous at higher densities. Stitched images in Supplementary Figure S1 do not show the through-plane density variation predicted by Bonnissel et al.^19^. At the scale of tens of micrometers (Fig. 1b), the microscope images show layers of flattened ENG particles whose preferred orientation increases with increasing density. Previous X-ray diffraction stuides^14,22,30,31^ state that the mean angle of graphite basal planes with respect to the sheet plane varies from $9^{\circ }$ to $20^{\circ }$ and that its change with sheet density is low at densities higher than $0.5\,\hbox {g\,cm}^{-3}$. Assuming that the the linear features in Fig. 1b correlate with the graphite basal planes, the observed angles are qualitatively consistent with the previous work. The highest-magnification images at a micrometer scale shown in Fig. 1c reveal thin features whose thickness and spacing decrease with increasing density. The decrease of thickness with increasing density suggests that the features are not crystalline because graphite crystals are not expected to undergo permanent thickness reduction under compression at pressures relevant to NGS. The achieved level of magnification prevents forming definitive conclusions, however, we interpret the thin features as clusters of cell walls whose porosity allows for their thickness to be reduced by the pressure during the forming process.

The images of sheet faces shown in Fig. 1d,e indicate that the surface structure changes from rough to smooth with increasing density. The boundaries of the ENG particles are visible in the low magnification image of the $0.55\,\hbox {g\,cm}^{-3}$ and $1.05\,\hbox {g\,cm}^{-3}$ sheets. At higher densities, the surface appears uniform at low magnifications, and signs of overlapping cell walls are apparent in the high magnification images.

For relating the structure of NGS to its material properties, it should be noted that crystalline graphite is highly anisotropic. The orientation of crystalline cell walls dictate the anisotropy of NGS. At high NGS densities, the cell walls are aligned parallel to the sheet plane, which leads to the properties closer to those of a graphite crystal. However, due to porosity, imperfect contact between the cell walls, crystal defects, and other imperfections, the properties never reach those of a graphite crystal.

### Material properties

**Figure 2:**
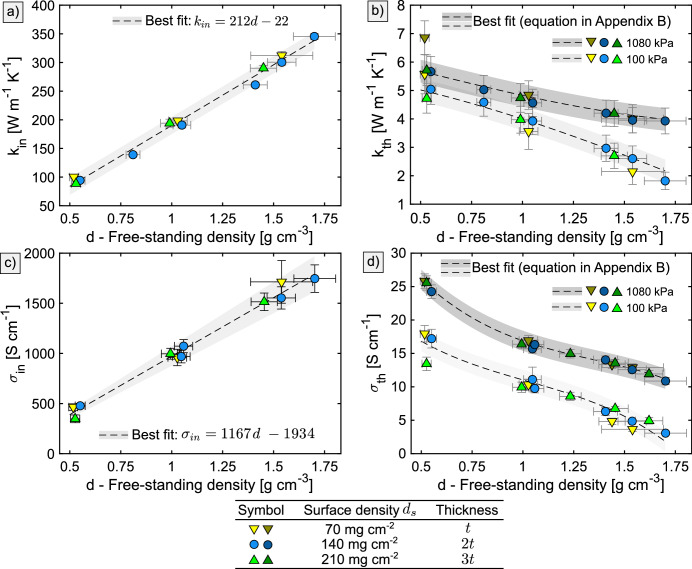
The **(a)** in-plane thermal conductivity $k_{in}$, **(b)** through-plane thermal conductivity $k_{th}$, **(c)** in-plane electrical conductivity $\sigma _{in}$, and **(d)** through-plane electrical conductivity $\sigma _{th}$ as a function of NGS density.

The measured in-plane thermal and electrical conductivities ($k_{in}$ and $\sigma _{in}$) increase linearly with density as is shown in Fig. 2a,c. In the through-plane direction, the thermal and electrical conductivites ($k_{th}$ and $\sigma _{th}$) decrease with density as can be seen in Fig. 2b,d. Both $k_{th}$ and $\sigma _{th}$ increase with through-plane compression pressure. For $k_{th}$, the increase is not significant at low densities, but becomes progressively higher with increasing density. For $k_{th}$, the increase is approximately constant over the entire measured density range. The experimental methods employed in this study are not suitable for measuring $k_{th}$ and $\sigma _{th}$ at pressures below 100 kPa due to the high and unpredictably variable contact resistance between sensors and samples. Attempts of low-pressure measurements indicated a steep decrease in $k_{th}$ and $\sigma _{th}$, but future measurements using novel methods are necessary to confirm or reject the observed trend. Measurement of $k_{in}$ at varying pressures did not reveal a pressure dependence, and the in-plane electrical conductivity $\sigma _{in}$ was measured in uncompressed state only.

Within the measured range of densities, pressures, and thicknesses, the thermal and electrical conductivities do not vary significantly with the sheet thickness, which can be supported by the overlap of the triangle and circular symbols in Fig. 2. The different sample thickness was achieved by using NGS at three surface densities ($d_s$) of 70, 140 and $210\,\hbox {mg\,cm}^{{-2}}$. Since stacks of NGS were measured in the through-plane direction, the insignificant variation with sheet thickness also suggests that the electric and thermal contact resistance at the sheet-to-sheet interfaces is negligible within the measured pressure range 100 kPa to 1080 kPa.

The low density of NGS results in high thermal diffusivity ranging from $230\,$ to ${270}\,{\hbox {mm}^2\hbox {s}^{-1}}$ in the in-plane direction ($\alpha _{in}$) and from ${1.5\,}$ to ${12}\,{\hbox {mm}^2\hbox {s}^{-1}}$ in the through-plane direction ($\alpha _{th}$). The plots of thermal diffusivity versus density are given in Appendix B.

The magnitude of thermal and electrical conductivity is dictated by the conductivity and orientation of the cell walls and the contact resistance between them. In the in-plane direction, the increase of $k_{in}$ and $\sigma _{in}$ with density arises from the decrease of cell wall misalignment and the decreased contact resistance due to improved contact quality or formation of new contact points. In the through–plane direction, the observed decrease of $k_{th}$ and $\sigma _{th}$ is contrary to the structure features seen in Fig. 1a,c. Closing of the slit-shaped pores and the decrease of the cell wall spacing with increasing density is expected to increase the through-plane conductivities. However, since the measured trend is opposite, the decrease in cell wall misalignment must be the dominating factor.

Besides the density and through-plane compression pressure, the material properties of NGS have been shown to be affected by the apparent density of ENG particles $d_{ENG}$^17,22^, flake size^44^, impurity content^14,45^, crystal defects^46^, rolling direction^14^, or heat treatment^14,47^. A comparison of the present thermal and electrical conductivities with the literature values^6,17,18,19,20,21,22,39^ is given in Supplementary Figures S2 and S3, but since the relevant parameters are varying or have not been reported, it is not possible to reliably explain the reasons for observed differences. Moreover, different measurement methods were used across the literature sources, which further broadens the list of possible sources of the discrepancies. Extensive future work is required to evaluate the sensitivity of NGS properties to the parameters listed above.

**Figure 3:**
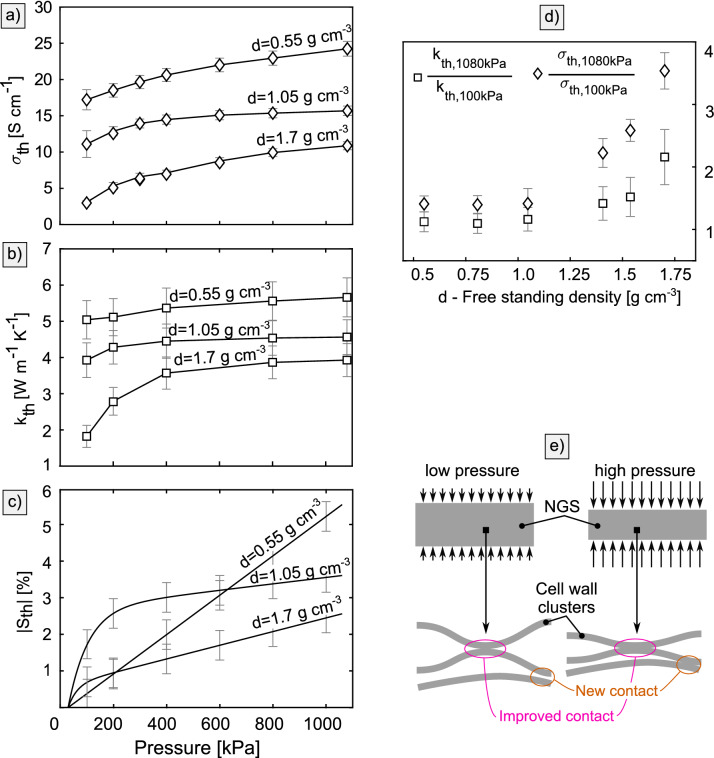
The pressure dependence of **(a)** through-plane electrical conductivity and **(b)** through-plane thermal conductivity with **(c)** through-plane compression strain. The relative increase of the through-plane thermal and electrical conductivities at 1080 kPa relative to 100 kPa is shown in **(d)**. Only the ${140}\hbox {mg\,cm}^{-2}$ samples are shown to improve plot clarity. A schematic of the change of NGS structure with through-plane compression is shown in **(e)**.

The detailed compression behavior of $k_{th}$ and $\sigma _{th}$ together with the through-plane stress-strain compression curves for three selected densities is shown in Fig. 3a–c. The change of $k_{th}$ with pressure is qualitatively proportional to the through-plane compression strain $S_{th}$. Both $k_{th}$ and $S_{th}$ of the ${0.55}\,\hbox {g\,cm}^{-3}$ sheet show a linear increase with pressure. At higher densities, $k_{th}$ and $S_{th}$ become progressively nonlinear with a higher rate of change at low pressures. The through-plane electrical conductivity $\sigma _{th}$ does not show the same proportionality to $S_{th}$ as $k_{th}$. The relative increase of $k_{th}$ and $\sigma _{th}$ at 1080 kPa with respect to the values at 100 kPa was plotted in Fig. 3d. The pressure sensitivity increases with increasing density, and $\sigma _{th}$ is more sensitive to pressure than $k_{th}$. The increase of $k_{th}$ and $\sigma _{th}$ with pressure is likely caused by the elastic deformation of cell walls and the improved contact quality between them, as is shown in Fig. 3e. Contrary to the forming process, no significant change in the misalignment of cell walls is expected.

**Figure 4:**
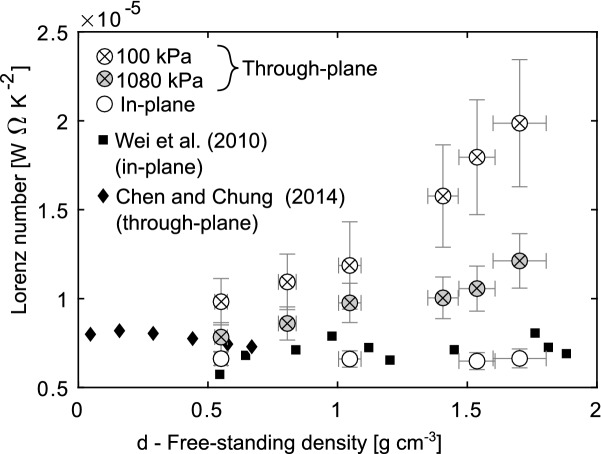
Lorenz number of NGS in the in-plane and through-plane directions as a function of density. Only the results for $140\,\hbox {mg\,cm}^{-2}$ samples are shown to improve plot clarity.

To verify the claim that NGS follows the Wiedemann–Franz law^21^, Lorenz number was calculated and plotted in Fig. 4 where it is seen to remain constant at $6.6 \times 10^{-6}\,\hbox {W}\Omega \,\hbox {K}^{-2}$ in the in-plane direction, but increase in the through-plane direction. The Wiedemann–Franz law is therefore concluded to be valid in the in-plane direction, but not in the through-plane direction.

**Figure 5:**
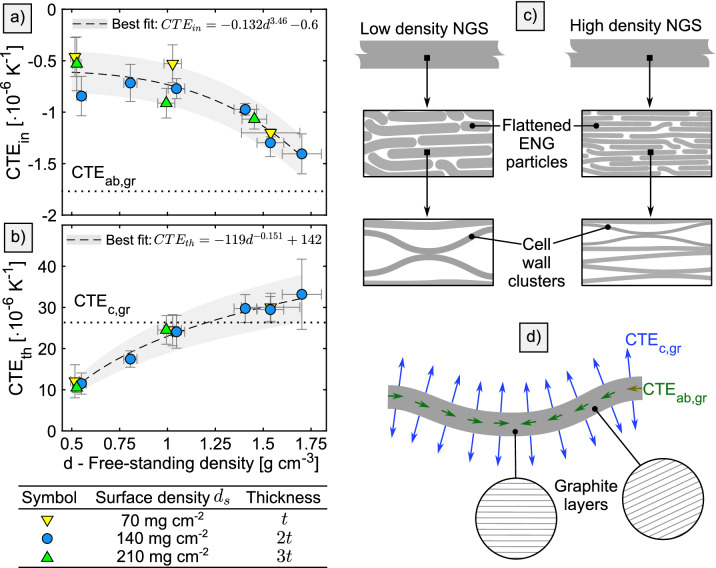
Results of CTE measurements in **(a)** in-plane and **(b)** through-plane directions. The relevant graphite crystal values $CTE_{ab,gr}$ and $CTE_{c,gr}$^41,42,43^ are demarcated by the dotted lines. In **(c)** a schematic structure of NGS is shown, and **(d)** illustrates the deformation of a single cell wall with increasing temperature.

The in–plane CTE of NGS ($CTE_{in}$) shown in Fig. 5a is low, negative, and its magnitude increases with density. CTE in the through-plane direction ($CTE_{th}$) is large, positive, and increases with density up to $33\,\times \,10^{-6}\,\hbox {K}^{{-1}}$ as is shown in Fig. 5b. The anisotropy and density dependence arises from the NGS structure, which was illustrated in Fig. 5c. At room temperature, the CTE of cell walls is $-1.8\,\times \,10^{-6}\,\hbox {K}^{{-1}}$ in the *ab* crystal directions ($CTE_{ab,gr}$) and $26\,\times \,10^{-6}\,\hbox {K}^{{-1}}$ in the *c* direction ($CTE_{c,gr}$)^41,42,43^. The deformation of a single cell wall with increasing temperature was outlined in Fig. 5d where the arrows symbolize the direction and magnitude of the dimension change. The CTE of NGS is dictated by the orientation and interaction of all cell walls. At high densities, $CTE_{in}$ approaches $CTE_{ab,gr}$ as expected based on the highly-oriented structure. At densities above ${1.2}\,\hbox {g\,cm}^{{-3}}$, $CTE_{th}$ appears to be higher than $CTE_{c,gr}$, which is contrary to the expectations. However, the measurement uncertainty prevents a definitive conclusion. It is likely that the expansion of air in the pores or the residual intercalation agent between graphite layers can cause an increase in $CTE_{th}$ beyond $CTE_{c,gr}$.

**Figure 6:**
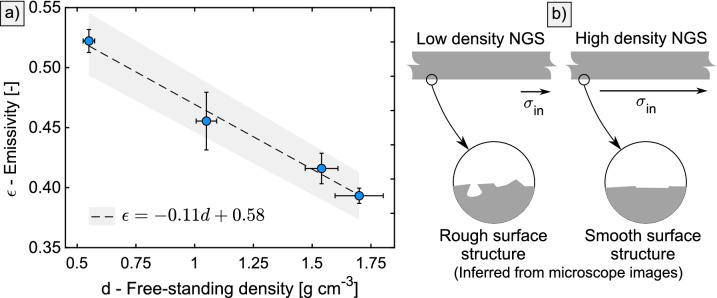
**(a)** The total emissivity of NGS versus the free-standing density, **(b)** an illustration of the change of the in-plane electrical conductivity $\sigma _{in}$ and surface structure with NGS density.

The total emissivity at faces of NGS for wavelengths ${2}\,{\upmu \hbox {m}}$ to ${26}\,{\upmu \hbox {m}}$ decreases with increasing density, from 0.52 at ${0.55}\hbox {g\,cm}^{{-3}}$ to 0.39 at ${1.7}\,\hbox {g\,cm}^{{-3}}$ as shown in Fig. 6a. The emissivity of graphitic materials has been previously related to the in-plane electrical conductivity $\sigma _{in}$ and surface roughness^49^. As shown in the simplified illustration in Fig. 6b, $\sigma _{in}$ increases with increasing density, while the surface structure changes from rough to smooth. Both of the factors contribute to the decrease in emissivity, the former via the Hagen–Rubens relation^49^, and the latter via an increased surface area and the small pores that act as radiation black bodies.

## Experimental methods

In-plane and through-plane thermal conductivities were measured using the transient plane source method (Hot Disk TPS 2500S) in the slab and one-dimensional modes, respectively. Electrical conductivity was measured using a four probe method with the Raytech Micro Junior 2 micro ohm meter. The compression behavior was measured in a mechanical tester with circular compression platens 50 mm in diameter and 3 kN load cell (Bose Electroforce 3300 Series II). CTE was measured using a thermomechanical analyzer (TA Instruments TMA Q400). Emissivity was determined from reflectivity measurements, which were performed in accordance with the Method C of ASTM E408-13 using an infrared reflectometer (Surface Optics Corporation 400T). All measurements were performed at room temperature except for CTE, which was determined in the range ${30}\,^\circ \hbox {C}$ to ${100}\,^\circ \hbox {C}$. The samples were prepared by calendering a low-density ${0.2}\,\hbox {g\,cm}^{{-3}}$ NGS into the desired density and thickness. NGS was supplied by Nano Carbon Technology CO. and its properties were fixed carbon content 99.27 %, $d_{ENG}$ ${4}\,\hbox {mg\, cm}^{-3}$, flake size composition 81 % larger than $300\,\upmu \hbox {m}$ (50 mesh, before exfoliation), intercalation agent H$_2$SO$_4$+H$_2$O$_2$+KMnO$_4$, exfoliation temperature $950\,^{\circ }\hbox {C}$, and exfoliation time 5 min. A number of samples was tested to capture the variability of the properties. For example, for the through-plane thermal conductivity, 20 samples were prepared for each of the measured densities, resulting in the total of 240 samples. A random combination of 20 samples was measured at least three times at each of the compression pressures, which resulted in the total of 222 measurements. The density of the samples was determined by measuring the dimensions and weight using an OHAUS AX124 scale and a Darson Instruments 0-1” analog micrometer. The samples for cross-section SEM images were prepared by impregnating NGS in epoxy resin, polishing, and removing the smeared flakes by plasma etching. FEI Nova NanoSEM microscope was used for imaging. The compression behavior measurements were reported elsewhere^15^. Details about the measurement methods, sample preparation, and uncertainty analysis were addressed at length in the supplementary document. The raw data, data processing scripts, microscope images in high resolution, and results in a tabular form are available in the supplementary dataset^36^.

### Supplementary Information

Supplementary Figure 1.

Supplementary Figure 2.

Supplementary Figure 3.

Supplementary Information 1.
