# Supplementary material for: Material properties and structure of natural graphite sheet
Source: Sci Rep. 2020 Oct 29;10:18672. doi: 10.1038/s41598-020-75393-y (PMC7596098; doi:10.1038/s41598-020-75393-y)
Supplement: Supplementary file 1 — Supplementary Figure 1. [file 41598_2020_75393_MOESM1_ESM.pdf]

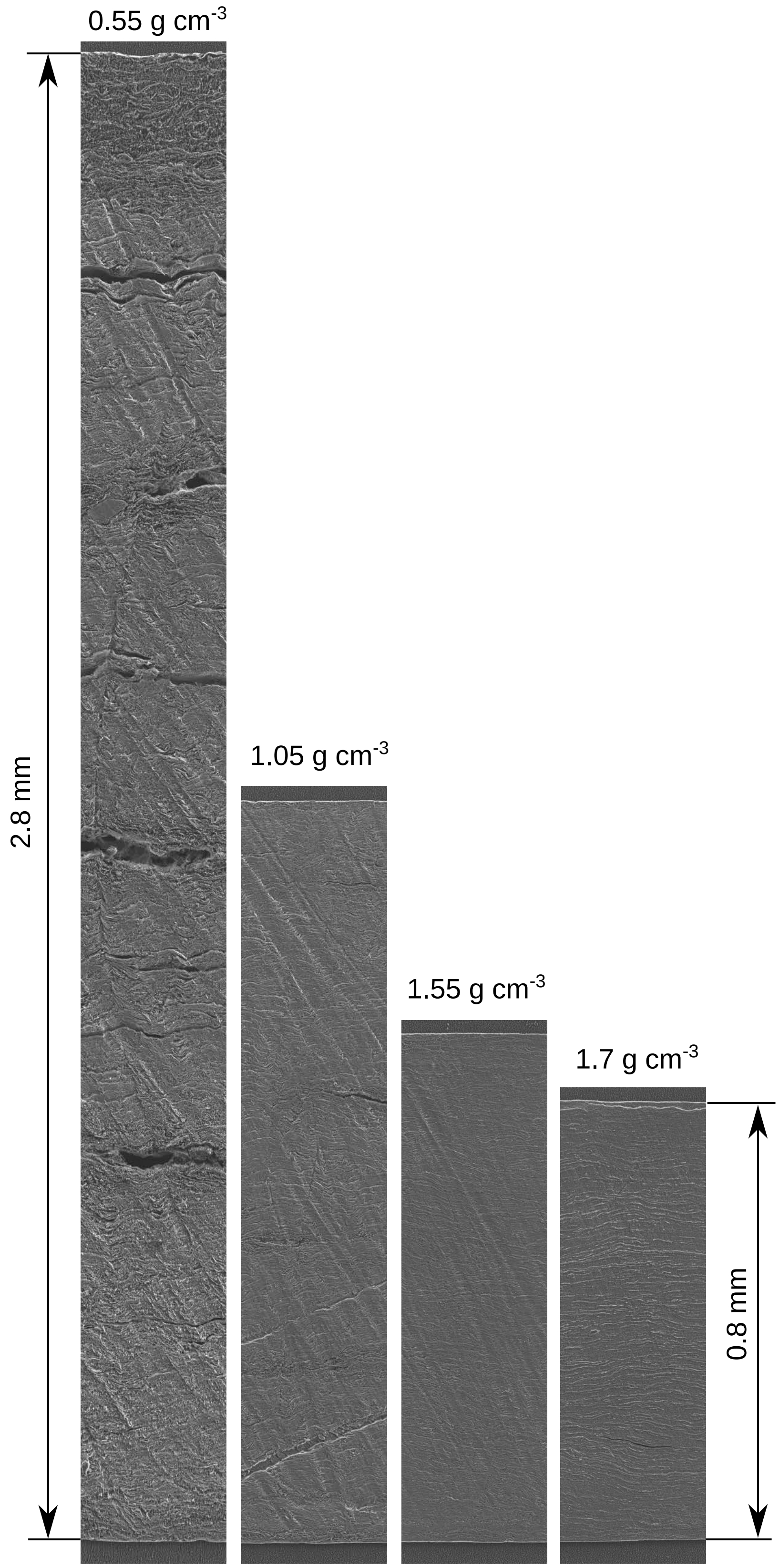

Figure S1 - Stitched microscope images of NGS cross-sections. The images were created by merging multiple raw microscope image files using Image Composite Editor software. High resolution images are available in the accompanying dataset.
