# Supplementary material for: Material properties and structure of natural graphite sheet
Source: Sci Rep. 2020 Oct 29;10:18672. doi: 10.1038/s41598-020-75393-y (PMC7596098; doi:10.1038/s41598-020-75393-y)
Supplement: Supplementary file 2 — Supplementary Figure 2. [file 41598_2020_75393_MOESM2_ESM.pdf]

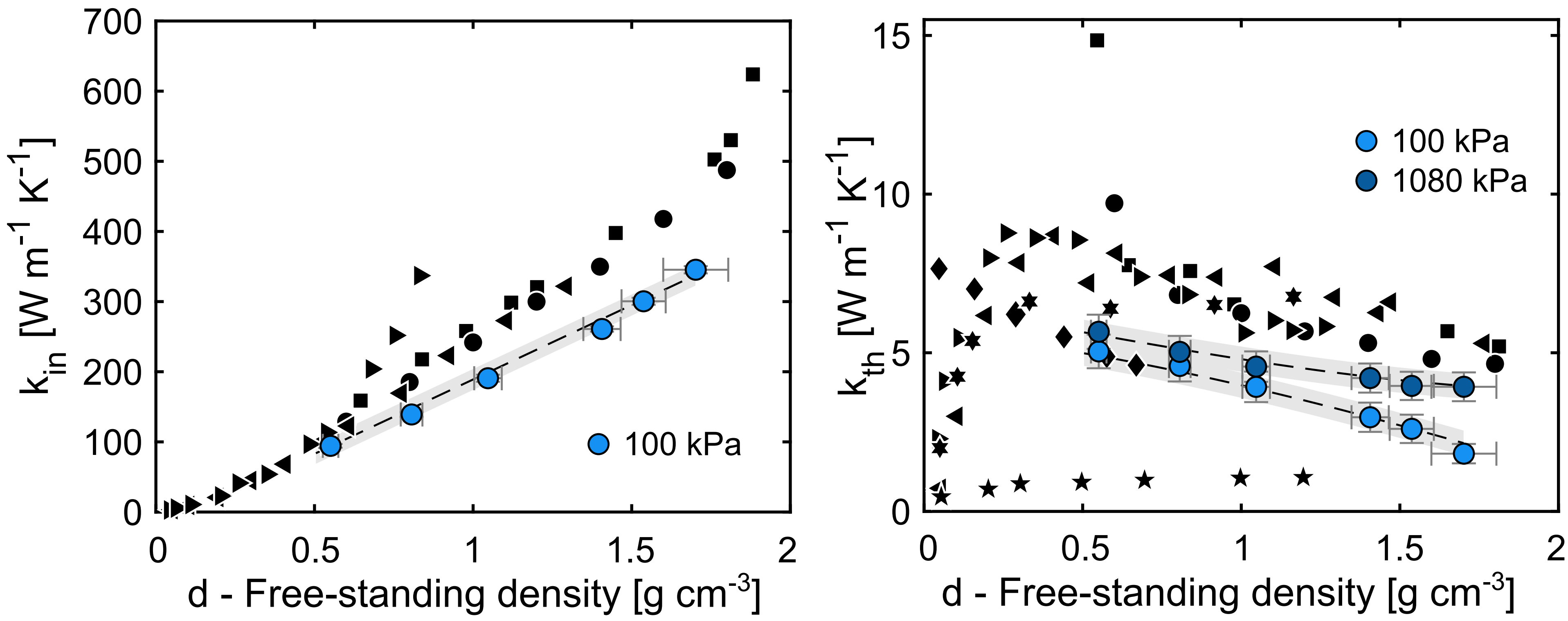

	Method	c_p [J kg^{-1} K^{-1}]	Pressure [kPa]	Thickness [mm]	d_{ENG} [mg cm^{-3}]	Fixed carbon content [%]	Flake size information
■ Wei et al. (2010)	LF	850	0	0.15 - 2	6.25	99.5	2 - 2.5mm (ENG particles)
● Liu et al. (2013)	LF	850	0	N/A	moderate'	N/A	N/A
◀ Bonnissel et al. (2001)	LF	850	0	N/A	1.59	N/A	0.25 mm diameter, few millimetre long' (ENG particles)
▶ Wang et al. (2011)	GHFM	734	N/A	5 - 12	5 - 6	100	N/A
♦ Chen and Chung (2014	GHFM	N/A	460	1.5 - 3.5	N/A	N/A	N/A
★ Afanasov et al. (2009)	LF	711	0	1.3 - 1.5	1	N/A	N/A
✶ Afanasov et al. (2009)	LF	711	0	1.3 - 1.5	2.5	N/A	N/A
●● Present work	TPS	729.3	100-1080	0.4 - 4.1	4	99	raw flakes 81% > 300μm

LF - Laser flash, GHFM - Guarded heat flow meter, TPS - Transient plane source

Figure S2. A comparison of the measured in-plane (k_{in}) and through-plane (k_{th}) thermal conductivity with the available literature data. The extended legend contains the specific heat capacity c_p, through-plane compression pressure, sheet thickness, apparent density of ENG particles d_{ENG}, fixed carbon content, and the available information about the raw graphite flakes. The present data are shown only for 140 mg cm^{-2} sheets, and the 70 and 210 mg cm^{-2} sheets (triangle symbols in Figure 2 in the main text) were hidden to improve clarity. The dashed lines are the best fits whose equations are available in Appendix B.
