# Supplementary material for: Material properties and structure of natural graphite sheet
Source: Sci Rep. 2020 Oct 29;10:18672. doi: 10.1038/s41598-020-75393-y (PMC7596098; doi:10.1038/s41598-020-75393-y)
Supplement: Supplementary file 3 — Supplementary Figure 3. [file 41598_2020_75393_MOESM3_ESM.pdf]

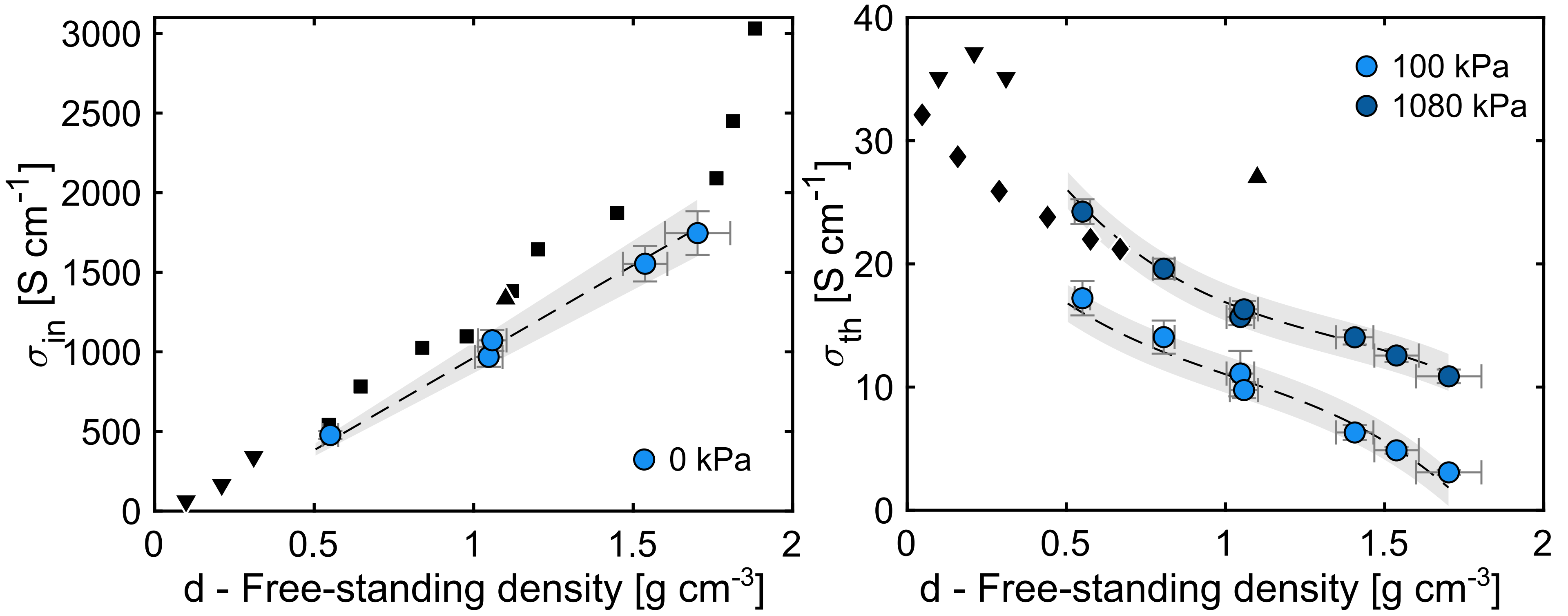

	Method	Pressure [kPa]	Thickness [mm]	d_{ENG} [mg cm^{-3}]	Fixed carbon content [%]	Flake size information
■ Wei et al. (2010)	Four probe	0	0.15 - 2	6.25	99.5	2 - 2.5mm (ENG particles)
▲ Luo and Chung (1996)	Four probe	0	3.1	N/A	95	N/A
▼ Celzard et al. (2000)	Four probe	0	20	7.5	N/A	N/A
◆ Chen and Chung (2014)	Four probe	460	1.5 - 3.5	N/A	N/A	2 - 4 mm (ENG particle length)
●● Present work	Four probe	100-1080	0.4 - 4.1	4	99.3	raw flakes 81% > 300µm

Figure S3. A comparison of the measured in-plane (σ_{in}) and through-plane (σ_{th}) thermal conductivity with the available literature data. The extended legend contains the measurement method, through-plane compression pressure, sheet thickness, apparent density of ENG particles d_{ENG}, fixed carbon content, and the available information about the raw graphite flakes. The present data are shown only for 140 mg cm^{-2} sheets, and the 70 and 210 mg cm^{-2} sheets (triangle symbols in Figure 2 in the main text) were hidden to improve clarity. The dashed lines are the best fits whose equations are available in Appendix B.
