# Supplementary material for: Material properties and structure of natural graphite sheet
Source: Sci Rep. 2020 Oct 29;10:18672. doi: 10.1038/s41598-020-75393-y (PMC7596098; doi:10.1038/s41598-020-75393-y)
Supplement: Supplementary file 4 — Supplementary Information 1. [file 41598_2020_75393_MOESM4_ESM.pdf]

Supplementary document
Material properties and structure of natural graphite sheet

Martin Cermak, Nicolas Perez, Michael Collins, Majid Bahrami

Contents

1 Sample preparation **2**

2 Density **3**

3 Microscope imaging **3**

4 Heat capacity **7**

5 Thermal conductivity and diffusivity **8**
5.1 Experimental method 8
5.1.1 In–plane direction 9
5.1.2 Through–plane direction 10
5.2 Data processing 11

6 Electrical conductivity **12**
6.0.1 In–plane direction 13
6.0.2 Through–plane direction 14

7 Coefficient of thermal expansion **16**

8 Emissivity **20**

9 Uncertainty analysis **21**
9.1 Sample dimensions and area 21
9.2 Density 22
9.3 Compression pressure 22
9.4 Thermal conductivity and diffusivity 23
9.5 Electrical conductivity 23
9.6 Coefficient of thermal expansion 23
9.7 Emissivity 23

1 Sample preparation

All samples in this work were prepared from a $0.2\,\mathrm{g\,cm^{-3}}$ NGS at three surface densities $70\,\mathrm{mgcm^{-2}}$, $140\,\mathrm{mgcm^{-2}}$ and $210\,\mathrm{mgcm^{-2}}$, which translates to the thicknesses of 3.5 mm, 7 mm and 10.5 mm. The sheets were cut and further compressed using a calendering machine shown in Figure SD1. The machine consists of two 93.5 mm cylinders that are kept at the set distance by a pneumatic system that imposes a 6.9 kN force. The calendered sheets were 100 mm wide, 300 mm long, and their density and thickness ranged from 0.5 to $1.7\,\mathrm{gcm^{-3}}$ and from 0.4 to 4.1 mm as shown in Figure SD2. For each of the measurements, samples of the desired shape and size were cut from the calendered sheets. When calendering the high-density sheets ($1.7\,\mathrm{gcm^{-3}}$), the upper cylinder visibly lifted, which implies that the capacity of the calendering machine was reached.

Using sheets with varying surface density allowed preparing samples with matching density but different thickness. For example, a 1.2 mm thick sample prepared from the $140\,\mathrm{mgcm^{-2}}$ sheet has the same density as 0.6 mm thick sample prepared from the $70\,\mathrm{mgcm^{-2}}$ sheet. Samples with multiple thicknesses were desirable to study the effect of thickness, and investigating the thermal and electrical contact resistance in stacks of sheets.

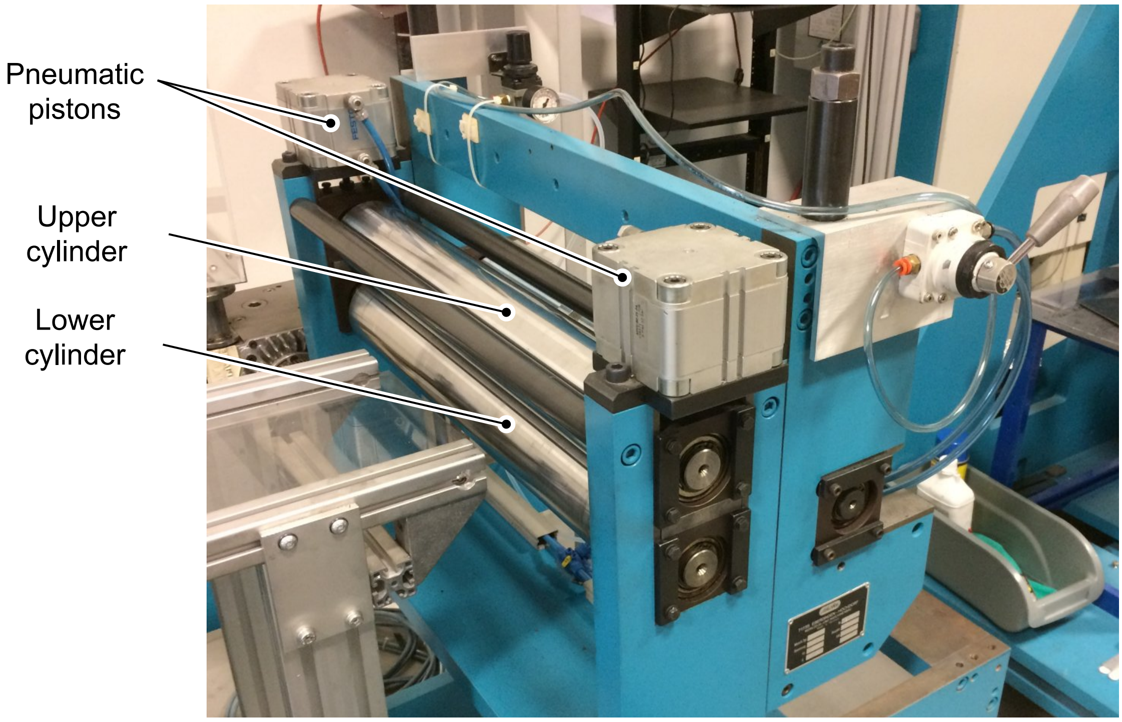

Figure SD1: An image of the calendering machine that was used for sample preparation. The image was provided by John Kenna of Terrella Energy Systems.

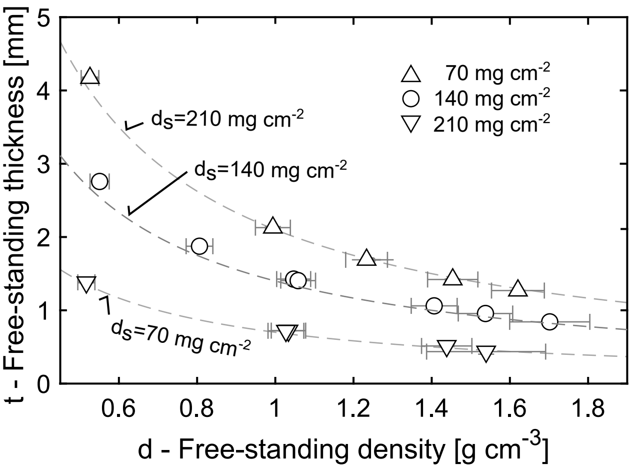

Figure SD2: The thickness and density of the sheets used in this study. The dashed line symbolize the theoretical relationship between the free-standing density d, thickness t, and surface density d_s.

2 Density

The density of the calendered sheets was determined by measuring the weight, thickness, and diameter of six discs that were cut using a 25 mm hole punch. The density was calculated as:

$$d = \frac{m}{tA} = \frac{m}{V}, \tag{1}$$

where m, t, A, and V are the disc mass, thickness, surface area, and volume, respectively. The weight was measured using an OHAUS AX124 scale and the dimensions by a Darson Instruments 0-1" 211-1313 analog micrometer. The uncertainty analysis is presented in section 9.2. The samples for measuring density were also used for measuring the through–plane electrical conductivity.

3 Microscope imaging

NGS samples at four densities ($0.55\,\mathrm{gcm^{-3}}$, $1.05\,\mathrm{gcm^{-3}}$, $1.55\,\mathrm{gcm^{-3}}$ and $1.7\,\mathrm{gcm^{-3}}$) were cut from the $140\,\mathrm{mgcm^{-2}}$ calendered sheets whose preparation was described in section 1. An illustration of the sample preparation method is shown in Figure SD3.

Four studied NGS samples were placed in a 30 mm diameter mold, which was then filled with an epoxy resin. Once cured, the epoxy resin blocks were polished using a Struers Tegramin-20 polisher. The polishing process involved grinding the surface of the block to expose the NGS samples, and polishing to produce a uniformly flat analytical surface. Grinding of the block was done using a diamond-impregnated abrasive disc with an alcohol-based lubricant. A series of progressively finer grades of abrasive were used and ranged from 220 to 1200 grit until a uniform cross-section was achieved. The surface was then polished using diamond suspensions ranging from 3 to 6 µm. Given the very soft nature of the material, finer suspensions were not necessary. After each step of the process, the blocks were cleaned with denatured alcohol and checked for defects using an inverted microscope. Once polished, a thin carbon coating (25 Å) was applied on the surface of the sample to prevent charging in the SEM microscope. The sample preparation was done by SGS Canada Inc., Burnaby, B.C., Canada. A photograph of the mounted and polished sample is shown in Figure SD4a.

Initial observations of the polished cross-sections revealed smearing of the graphite layers as can be seen in Figure SD5. To expose the true cross-section, oxygen plasma etcher was used as illustrated in Figure SD3. The etching was performed at 280 mTorr pressure, the power was 300 W, and the total etching time was 50 min. The conductive coating was renewed after the plasma etching step.

After the plasma etching step, bright artifacts were discovered on the observed surface as shown in Figure SD6d. As shown in Figures SD6a and SD6b, it is assumed that the artifacts are a result of non-volatile contaminants that cannot be removed by plasma etching. Since the artifacts are present on both NGS and on the mounting resin (SD6c), the contaminants are most likely the

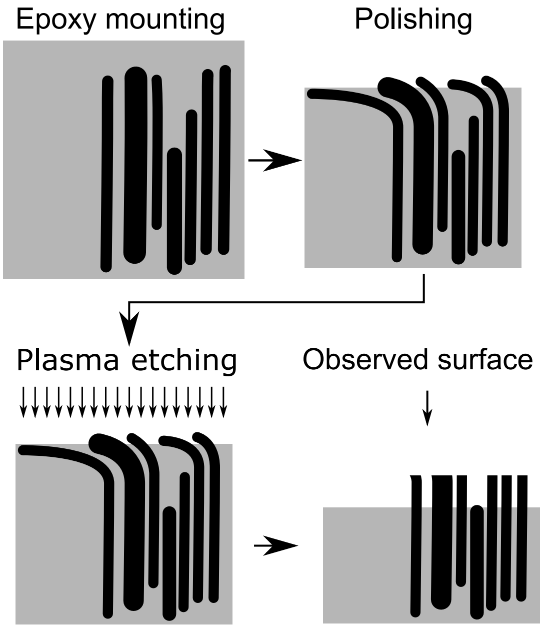

Figure SD3: Illustration of the sample preparation method.

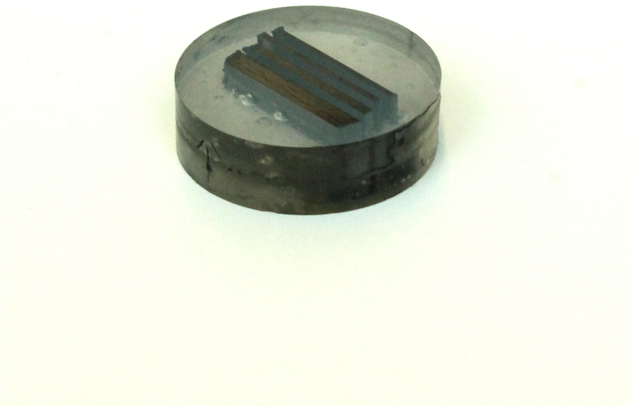

Figure SD4: Photograph of four NGS samples mounted in an epoxy cylinder.

residue particles from the polishing process. The structure of NGS was visible despite the artifacts and no further sample processing was done. It is likely that a sonication step can remove the non-volatile particles.

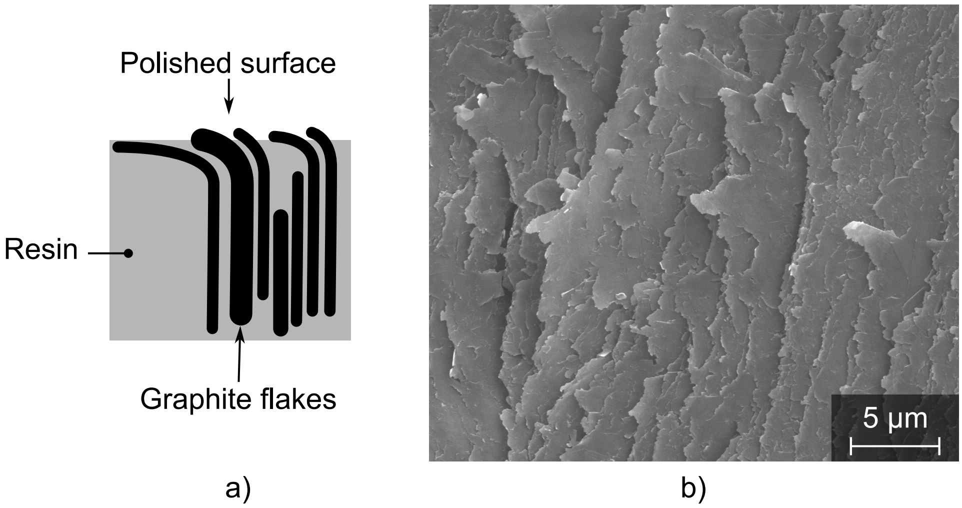

Figure SD5: SEM image of smeared surface after polishing (right) and a simplified illustration of the smeared structure (left).

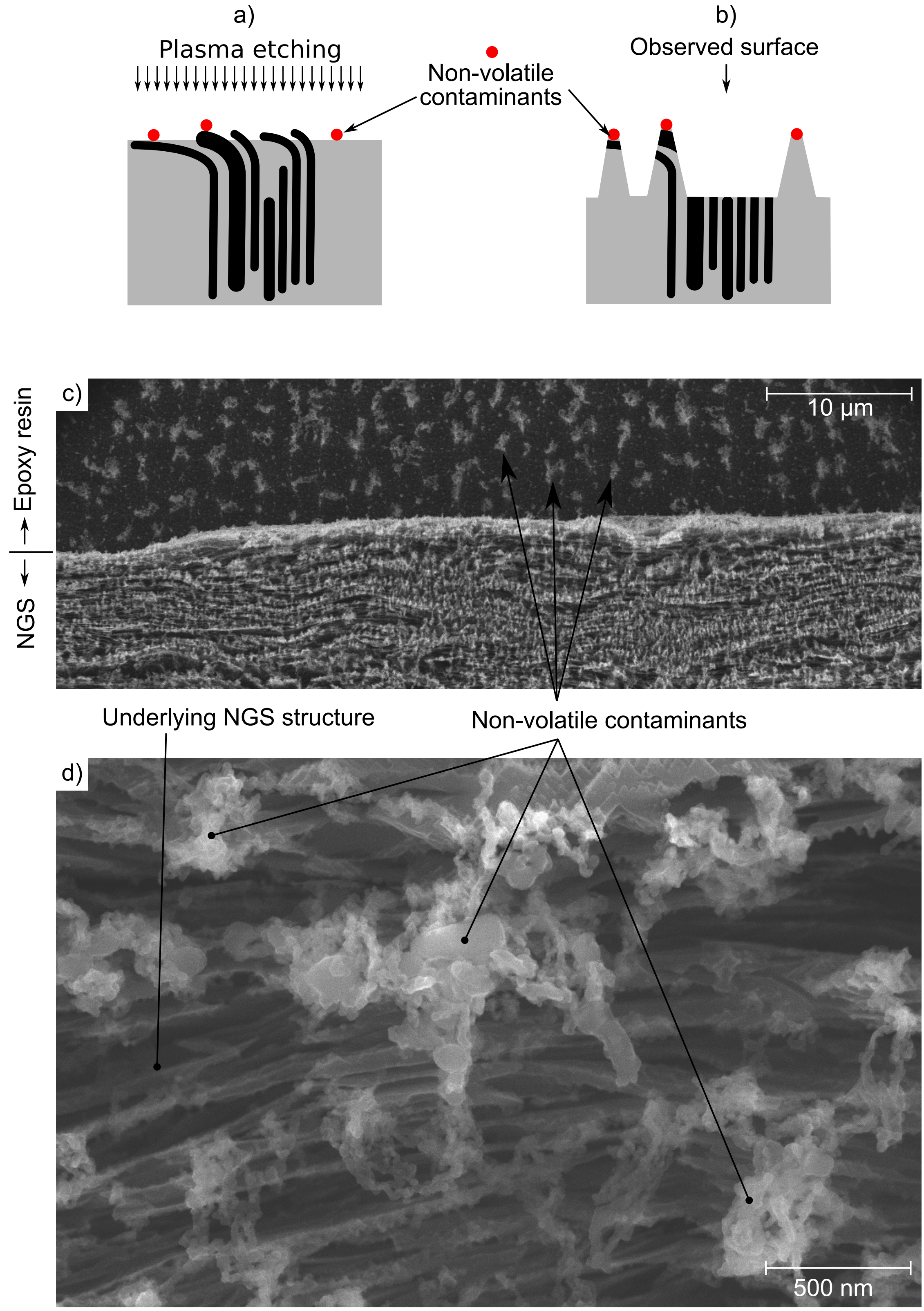

Figure SD6: a,b) A schematic visualization of the formation of image artifacts due to non-volatile contaminants, and c,d) microscope images showing the artifacts.

4 Heat capacity

In this work, the specific heat capacity of NGS was assumed to be equal to that of pure graphite because the fixed carbon content of the NGS used in the present study was 99.27 % and the typical value is large than 95 %. The base for determining the heat capacity of NGS was the relationship by Spencer [SD15] reported in [SD10], which is:

$$c_{m,gr} = -5.293 + 58.998 \times 10^{-3}T - 43.225 \times 10^{-6}T^2 + 11.51 \times 10^{-9}T^3\ [\mathrm{J\,mol^{-1}\,K^{-1}}], \quad (2)$$

where $c_{m,gr}$ is the molar heat capacity of graphite and T is the temperature in Kelvins. Using the molecular weight of carbon $M_{W,C} = 12.011\,\mathrm{g\,mol^{-1}}$ the specific heat capacity (per unit mass) is:

$$c_{p,gr} = -440.68 + 4.91T - 3.6 \times 10^{-3}T^2 + 9.58 \times 10^{-7}T^3\ [\mathrm{J\,kg^{-1}\,K^{-1}}]. \quad (3)$$

The data predicted by Equation 3 are compared to the experimental data by Wang et al. [SD17] in Figure SD7. It can be seen that the polynomial relationship by Spencer [SD15] predicts slightly higher values in comparison with the experimental data for NGS. However, at room temperature the difference is low and therefore the relationship by Spencer [SD15] was considered valid, and the specific heat capacity of NGS in this work was determined by evaluating Equation 3 at 25 °C, which resulted in the value of 729.3 $\mathrm{J\,kg^{-1}\,K^{-1}}$.

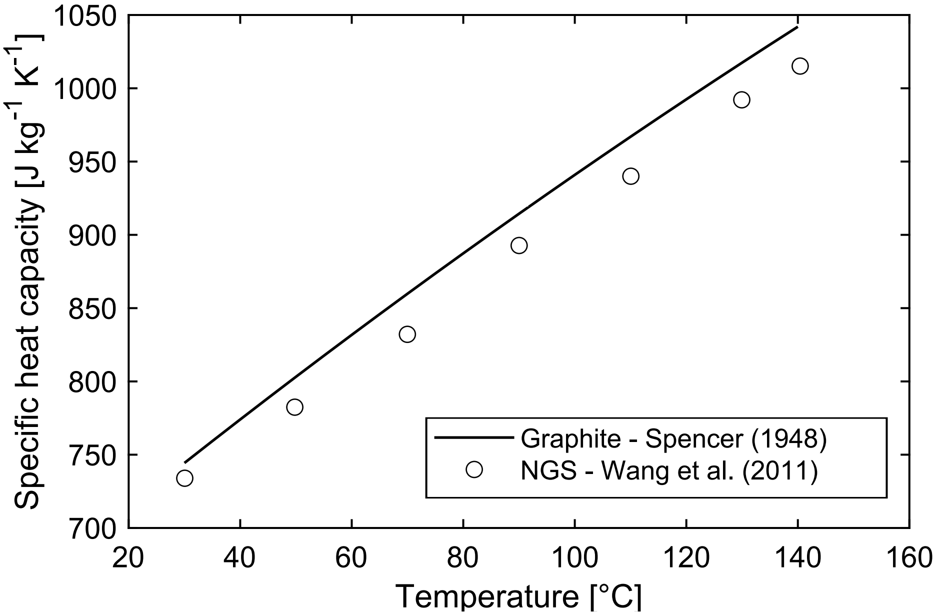

Figure SD7: The specific heat capacity of graphite (Spencer [SD15], solid line) and NGS (Wang et al. [SD17], circles) as a function of temperature.

Volumetric heat capacity c_v is required for the measurement of thermal conductivity and diffusivity using the TPS method. It is defined as:

$$c_v = dc_p, \quad (4)$$

where c_v is the volumetric heat capacity, d is density of the material, and c_p is the specific heat capacity (per unit mass).

Since the thickness of NGS reduces significantly under compression, the change of density needs to be considered when evaluating the volumetric heat capacity of NGS at a compressed state. The density in the free-standing state d is:

$$d = \frac{m}{tA}, \quad (5)$$

where m is the mass of the sample, t is the relaxed thickness, and A is the sample area. Density at a compressed state d_p is:

$$d_p = \frac{m}{t_pA}, \quad (6)$$

where t_p is the thickness under compression. The thickness under compression can be calculated based on the strain at a given pressure S_{th} as:

$$t_p = t - S_{th}t \tag{7}$$

Combining equations 5, 6, and 7 yields:

$$d_p = \frac{m}{tA(1 - S_{th})} = \frac{d}{1 - S_{th}} = K_d d, \tag{8}$$

where the density correction factor K_d was defined as:

$$K_d = \frac{1}{1 - S_{th}} \tag{9}$$

The volumetric heat capacity under compression is:

$$c_{v,p} = K_d d c_p = K_d c_v \tag{10}$$

The density correction factor K_d for four measured densities is shown in Figure SD8. The values for other densities were calculated using linear interpolation.

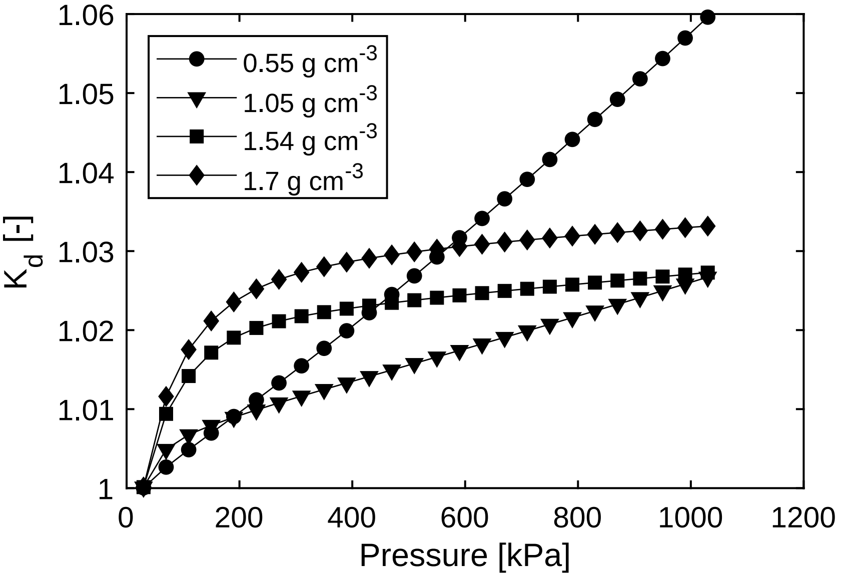

Figure SD8: The density correction factor of the volumetric heat capacity as a function of the pressure

5 Thermal conductivity and diffusivity

5.1 Experimental method

All the measurements in this study were performed using the Hot Disk TPS 2500S machine, which is a commercial implementation of the transient plane source method [SD6, SD7]. The method uses a sensor in the form of a nickel spiral. During the measurement the sensor is inserted between two identical samples, electric current is passed through it, and the generated heat penetrates the samples. The temperature increase of the sensor is recorded by measuring the current and voltage, and correlating it to the known resistivity of nickel. Based on the shape of the temperature-versus-time curve, the thermal properties can be determined. The TCR at the sensor-sample interface is excluded by discarding the initial section of the temperature-versus-time curve. An extended description of the measurement can be found in [SD4].

Pressure was imposed on the samples by manually turning a wheel attached to a threaded rod that lifts the sample table. The compression force was monitored by the Mark-10 M5-500 Series 5

force gauge. The measurement method and the sample size varied for the in–plane and through–plane directions. All the measurements were performed at 25 °C.

While the Transient Plane Source method is standardized in ISO 22007-2, it was not possible to fully comply with it. The fine-tuned data analysis method within the proprietary software deviates from the standard. Using the fine-tuned method was suggested by the device manufacturer. The one-dimensional mode that was used for the through–plane measurements was not included in ISO 22007-2.

5.1.1 In–plane direction

For the in–plane direction, the slab mode was used. 7 cm by 7 cm square samples were cut from the calendered sheets described in section 1. The measurement configuration consisted of the sensor, sample, insulation, and pressure spreader as shown in SD9a. The sensor radius was 3.189 mm for all the in–plane measurements except for the samples at the lowest density and high thickness. In this case, the large thickness required using the 6.403 mm sensor to satisfy the slab mode criterion $0.03125 < h/a < 0.79836$ given in [SD1], where h is the sample thickness and a is the sensor radius. The heating power was in the range 0.4 to 1.8 W to achieve the total temperature increase given in [SD1]. The measurement time, which should be chosen so that the heat penetration depth is less than the available probing depth, was always set to the low limit of the machine (1 s) due to the high in–plane thermal diffusivity of NGS. Optimal measurement conditions would require lower measurement times or larger samples. The latter was not possible due to the limited size of the test section. 12 sample sets with varying density and thickness were used for the measurements. Each of the sample sets contained at least five samples. Three to five measurements of two randomly selected samples were performed for each sample set, resulting in the total of 48 measurements.

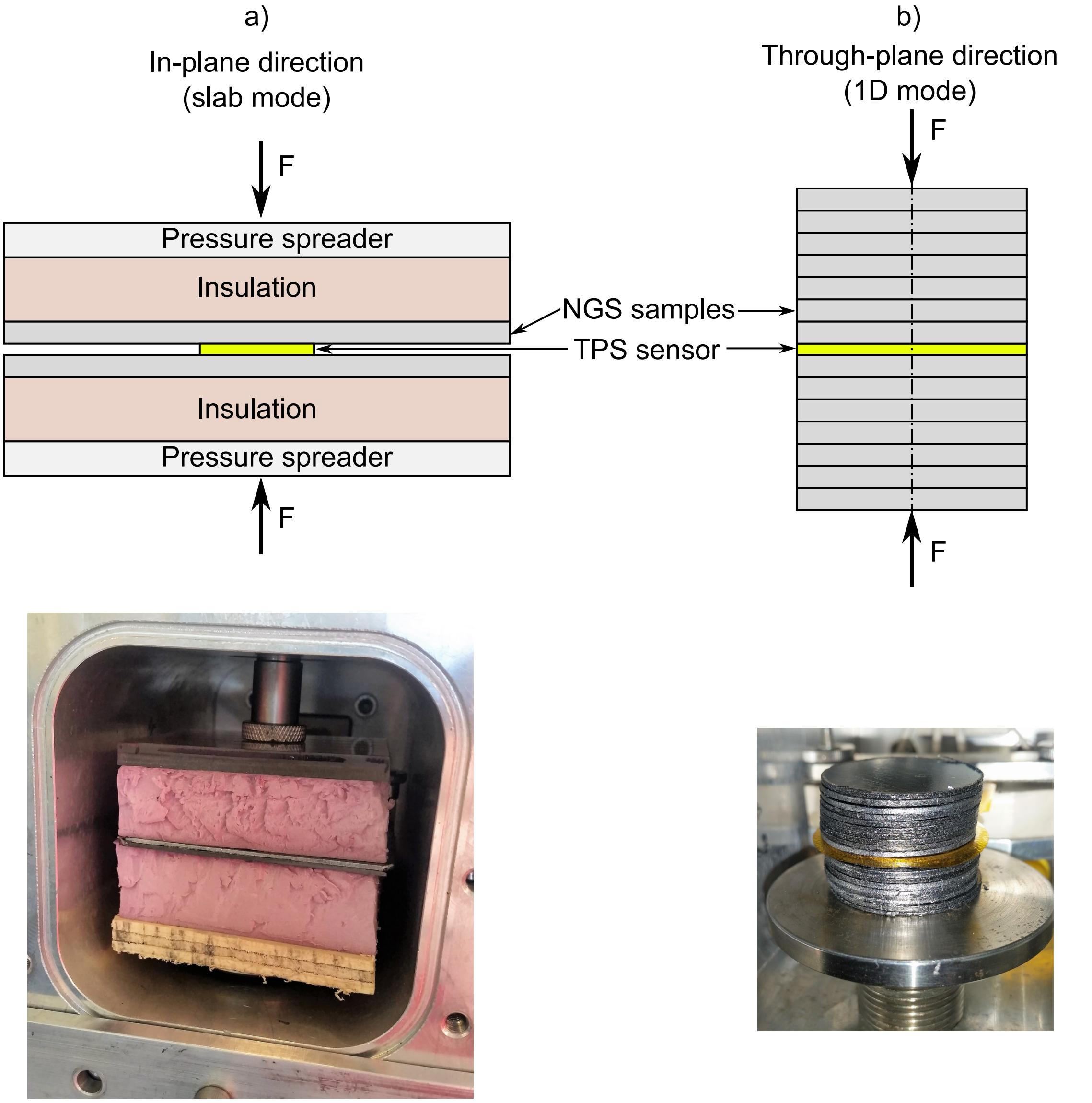

Figure SD9: Schematics (top) and photography (bottom) of the thermal conductivity measurement

5.1.2 Through–plane direction

To measure the through–plane thermal properties, the one-dimensional mode was used. It requires the samples to match the sensor size, and therefore circular samples 32 mm in diameter were cut from the calendered sheet using a hole punch, and the measurements were performed using the 29.2 mm sensor. The difference between the sensor and sample diameters was within the requirements given in Ref. [SD1]. Stacks of sheets with the total height ranging from 6.7 to 27 mm were measured at pressures of 100 kPa, 200 kPa, 400 kPa, 800 kPa and 1080 kPa. Measurements at no compression were attempted, however, the repeatability was low and the results were not included in this text. The measurement time and the heating power were fixed to 2 s and 3 W, respectively. In all cases the penetration depth was smaller than the total height of the stack. 12 sample sets with varying density and thickness were used for the measurements. Each of the sample sets contained at least twelve samples. Measured stacks were assembled from a random combination of the samples within the sample set. For each of the sample sets the measurements were repeated at least three times, resulting in the total of 222 measurements.

Measurement of the through–plane properties of a single sheet was not possible as the required measurement times are much lower than the low limit of the machine. Since stacks of sheets were measured, the sheet-to-sheet TCR is inherently included in the value of the through–plane thermal conductivity and diffusivity. Neither the TPS nor the guarded heat flow meter method are capable of deconvoluting of the sheet-to-sheet TCR. The importance of the sheet-to-sheet TCR can be estimated by comparing the results for stacks of sheets at different thicknesses. Figure SD10 illustrates the theory employed to judge the importance of the sheet-to-sheet TCR. The surface density of the sheet dictates its thickness at the given density. A varying number of interfaces per stack height can be achieved by using stacks of low and high surface density sheets. If the sheet-to-sheet TCR is significant, the stack with more interfaces will show lower effective thermal conductivity. In practice, it was not possible to manufacture sheets at the same density because the calendering process did not allow a fine control of the final sheet thickness. Therefore, the sheets prepared for this measurement had a comparable but not identical density, as can be seen by the horizontal distance between the circular and triangular symbols in the result (Figure 2 in the main document). Since the results do not show the vertical stacked pattern predicted for significant sheet-to-sheet TCR (Figure SD10 bottom-left), the measured thermal conductivity is deemed to be that of the bulk material, and the sheet-to-sheet TCR is considered negligible. The conclusion is in agreement with the work by Smalc et al. [SD14].

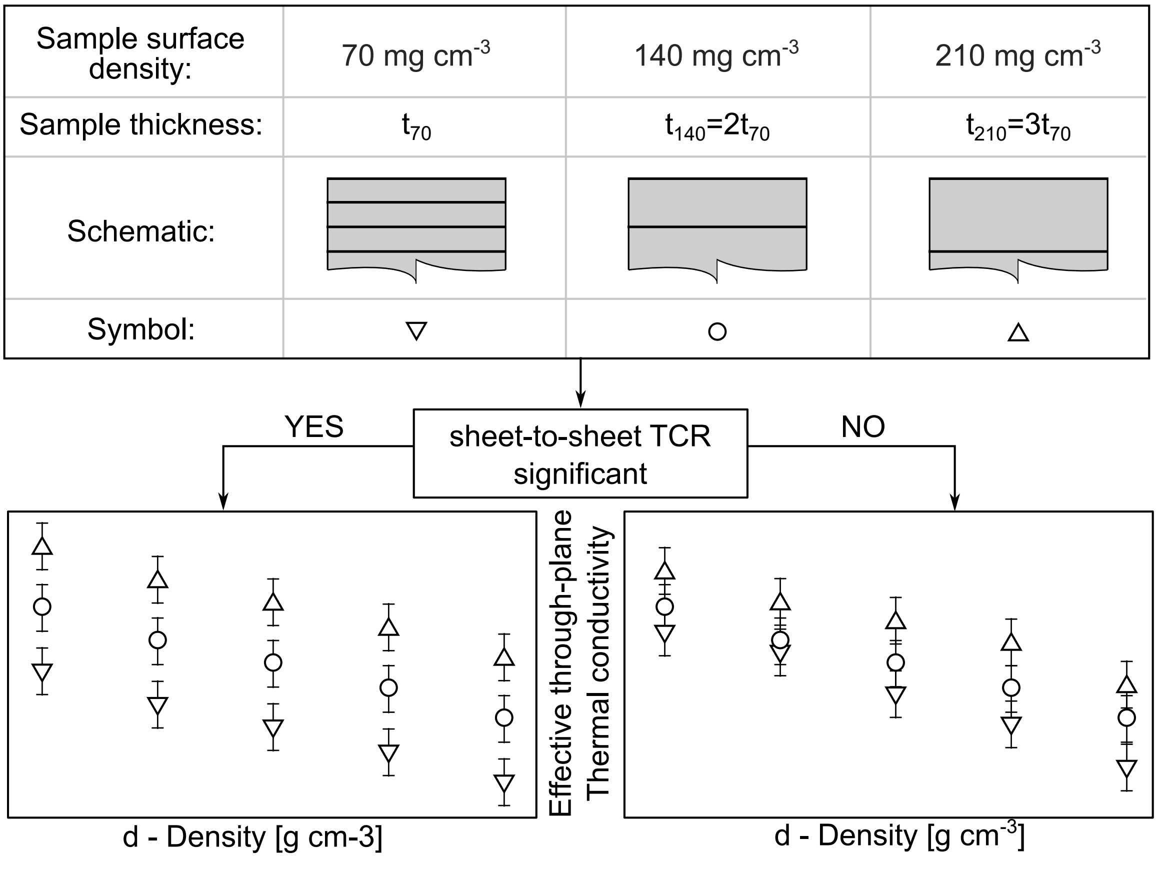

Figure SD10: Illustration of the theory used for evaluating the importance of the sheet-to-sheet TCR

5.2 Data processing

The data processing was done using the Hot Disk Thermal Constants Analyzer 7.3.12 software. While it is generally possible to determine the volumetric heat capacity using the TPS method, the modes used in this study required inputting it. The value of the volumetric heat capacity was calculated by multiplying the specific heat capacity of 729.3 $\mathrm{J kg^{-1} K^{-1}}$ and the density. The density

was corrected for the change with pressure as described in section 4. Both the raw data files and the implementation of data processing in Matlab are available in [SD3].

6 Electrical conductivity

The measurements were performed at 25 °C using a Raytech Micro Junior 2 micro ohm meter that uses the four probe method to measure the resistance of the samples of interest. The resistivity was calculated from the resistances based on the fundamental equation:

$$\rho = \frac{R}{t} A, \tag{11}$$

where ρ is resistivity, R is the electrical resistance, t is the sample thickness, and A is the sample cross-section. The measured resistance is a combination of the bulk resistance R_b and the probe-to-sample electric contact resistance $R_{ECR,p-s}$ as is shown on the left side of Figure SD11. To deconvolute the contact resistance at the probe-sample interface, the two-thickness method is typically used as illustrated in Figure SD11. The bulk resistance R_b can be expressed in terms of resistivity and

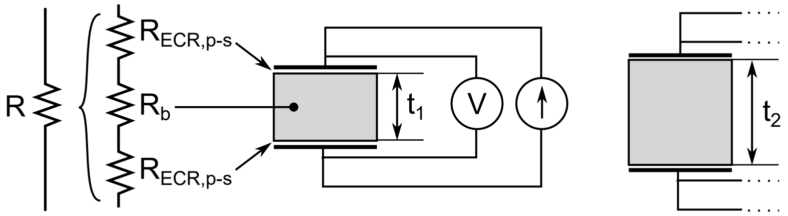

Figure SD11: Illustration of the two-thickness method for measuring the electrical resistivity.

sample dimensions as:

$$R_b = \rho \frac{t}{A}, \tag{12}$$

and the total measured resistance is:

$$R = \rho \frac{t}{A} + 2R_{ECR,p-s}. \tag{13}$$

Evaluating Equation 13 for two thicknesses t_1 and t_2 and subtracting the two equations yields:

$$R_2 - R_1 = \rho \frac{t_2}{A} - \rho \frac{t_1}{A} + 2R_{ECR,p-s} - 2R_{ECR,p-s}, \tag{14}$$

which simplifies to:

$$R_2 - R_1 = \rho \frac{t_2 - t_1}{A}, \tag{15}$$

and the resistivity of the measured sample can be calculated as:

$$\rho = \frac{(R_2 - R_1)A}{t_2 - t_1} \tag{16}$$

The electrical conductivity can be calculated as the inverse of resistivity. If necessary the contact resistance $R_{ECR,p-s}$ can be calculated by using the resistivity value in Equation 13 as:

$$R_{ECR,p-s} = \frac{R}{2} - \frac{\rho t}{2A}. \tag{17}$$

In this study, the two-thickness method was extended by measuring more than two thicknesses. The accuracy of the $\frac{R_2-R_1}{t_2-t_1}$ term can be improved by substituting it with the slope of the linear fit of the resistance versus thickness data points. Due to the vast difference between the in–plane and through–plane properties, the measurement method and sample shape varied for the two directions. All the raw data files and the implementation of data processing methods can be accessed in [SD3]. The principle of the measurement method is comparable to ASTM C611-98, but the standard could not be fully followed. An extended description of the measurement can be found in [SD4].

6.0.1 In–plane direction

For the in–plane measurements, a strip 210 mm long and 9 to 15 mm wide was cut for each of the eleven measured sheets. The ohm meter terminals were attached to the samples using clips in a way showed in Figure SD12. Each sample was measured at four nominal voltage terminal distances L_V of 50 mm, 100 mm, 150 mm and 200 mm that were set by locking a caliper at the given distance, attaching it to the sample, and clipping the terminals next to the upper jaws. For each of the measured distances, the measurement was repeated at least three times resulting in the total of 168 ohm meter measurements. Before each of the repetitions, the clips were removed and re-attached again. The measurement current was 1 A. One strip was manufactured for each of the measured sheets,

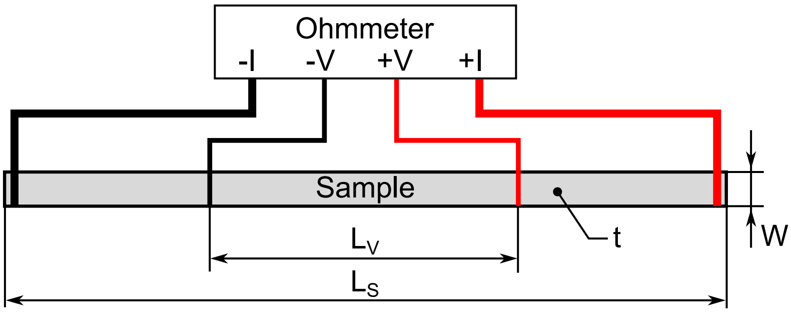

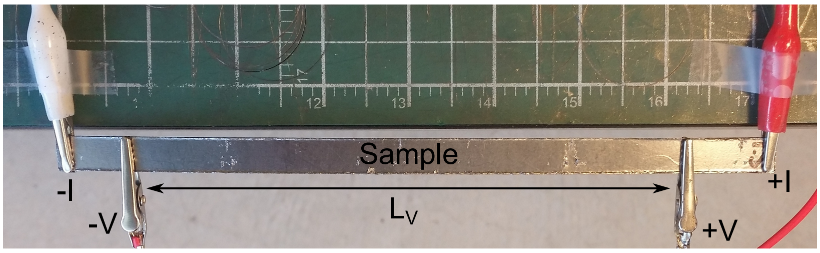

Figure SD12: A scheme (top) and a photograph (bottom) of the in–plane electrical conductivity measurement. The length, width, and thickness of the sample are marked as L_S, W, and t, respectively. +I and -I are the current terminals and +V and -V are the voltage terminals. The distance between the voltage terminals is L_V.

For calculating the in–plane resistivity ρ_{in}, the general Equation 11 was modified to reflect the measurement configuration shown in Figure SD12 as:

$$\rho_{in} = \frac{R}{L_V} A_{in}, \tag{18}$$

where R is the measured resistance, L_V is the distance between the voltage terminals, and A_{in} is the cross-section area for the in –plane measurements, which is defined as:

$$A_{in} = Wt, \tag{19}$$

where W and t_s are the sample width and thickness, respectively. For each of the samples, a line was fit thought the data points to obtain the slope a as illustrated in Figure SD13. The fraction on the right side of Eq. 16 corresponds to the slope of the fitted line a and Eq. 18 can be therefore rewritten to:

$$\rho = aA_{in}. \tag{20}$$

The slope of the line a was obtained using the *fit* function in Matlab.

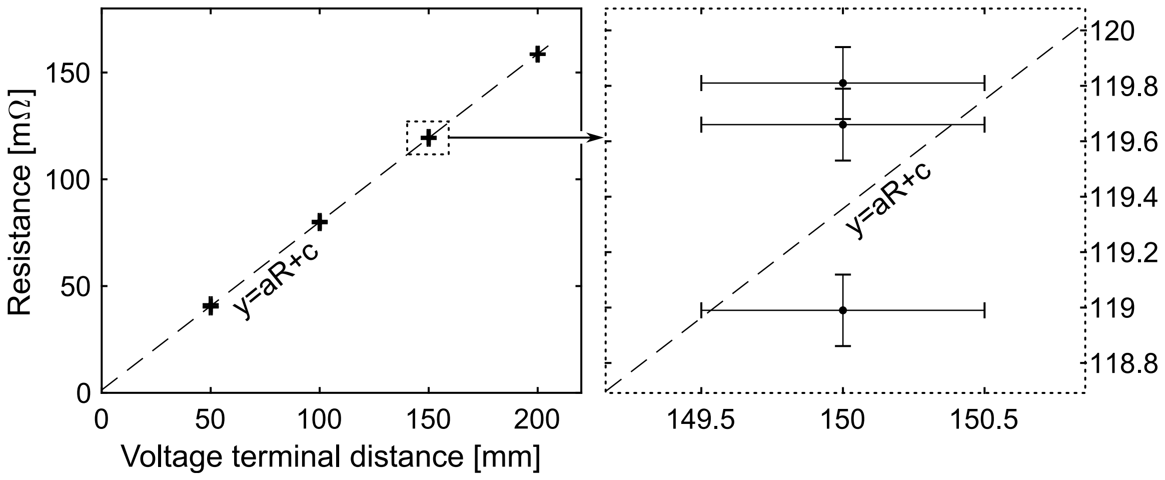

Figure SD13: The measured data for the $1\,\mathrm{g\,cm^{-3}}$ $140\,\mathrm{mg\,cm^{-2}}$ sample. Left: the resistance data for the four measured voltage terminals distances. Right: a detail of the 150 mm terminal distance showing the variation of the data points and the measurement uncertainty.

6.0.2 Through–plane direction

For the though–plane direction, disc-shaped samples 24.7 mm in diameter were used and measured between gold-plated copper probes at pressures ranging from 100 to 1080 kPa as shown in Figure SD14. The compression force was imposed using the same device as in the measurements of thermal conductivity (section 5). The measurement procedure consisted of mounting a stack of randomly selected samples between the probes, collecting the resistance readings at increasing values of pressure, and repeating the measurement for at least two more random combinations of samples within a sample set. 16 sample sets were prepared, each containing at least 20 samples. The total number of ohm meter measurements was 938.

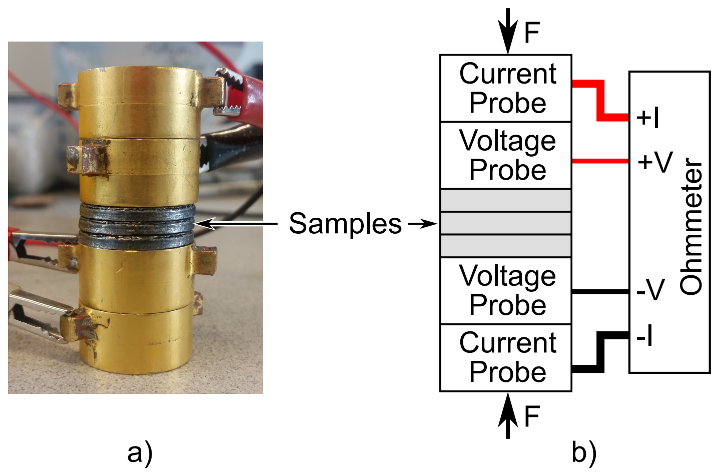

Figure SD14: a) a photograph and b) a scheme of the through–plane electrical conductivity measurement. The compression device is not shown for clarity.

In comparison with other materials such as metals, for which samples for the two-thickness method can be prepared by machining, preparing NGS samples at varying thickness and matching density requires the ability to control the compression in the forming process at high accuracy. At the sheet thickness less than 1 mm, a 0.1 mm change in thickness can results in a 20 % increase in density. In this work, the accuracy required to prepare the samples for the two-thickness method was not achievable. Using the two thickness method with two single-body samples was therefore rejected, and stacks of samples were measured instead. This configuration introduces additional

sample-to-sample (or sheet-to-sheet) contact resistances that must be accounted for.

As illustrated in Figure SD15, when a stack of sheet is measured, the total resistance R is a sum of the NGS bulk resistance R_b, probe-to-sheet contact resistance $R_{ECR,p-s}$, and the sheet-to-sheet contact resistance $R_{ECR,s-s}$. For the number of sheets n_s the total resistance is:

$$R = n_s R_b + 2R_{ECR,p-s} + (n_s - 1)R_{ECR,s-s}. \tag{21}$$

The bulk resistance R_b can be expressed as:

$$R_b = \rho_{th,b} \frac{t_s}{A_{th}} \tag{22}$$

where $\rho_{th,b}$ is the bulk through–plane resistivity, t_s is the sheet thickness, and A_{th} is the sample cross-section area relevant to the through–plane measurements. Substituting Equation 22 into 21 and rearranging the terms yields:

$$R = \underbrace{\left(\rho_{th,b} \frac{t_s}{A_{th}} + R_{ECR,s-s}\right)}_{a} n_s + \underbrace{2R_{ECR,p-s} - R_{ECR,s-s}}_{c}, \tag{23}$$

where the equation of the line $R = an_s + c$ outlined in Figure SD15 can be identified. The slope of the line a is:

$$a = \rho_{th,b} \frac{t_s}{A_{th}} + \frac{r_{ECR,s-s}}{A_{th}}, \tag{24}$$

where specific contact resistance $r_{ECR,s-s}$ was used instead of $R_{ECR,s-s}$. Rearranging Equation 24 yields:

$$\frac{aA_{th}}{t_s} = \rho_{th,b} + \frac{r_{ECR,s-s}}{t_s}, \tag{25}$$

where the first term corresponds to the effective resistivity $\rho_{th,eff}$ and the equation can be therefore rewritten as:

$$\rho_{th,eff} = \rho_{th,b} + \frac{r_{ECR,s-s}}{t_s}. \tag{26}$$

It can be seen that the effective resistivity is a combination of the bulk resistivity of NGS, specific contact resistance at the sheet-to-sheet interface, and the thickness of the sheet. The contribution of the contact resistance decreases with increasing sheet thickness, which can be explained by fewer interfaces per unit length.

The significance of the sheet-to-sheet contact resistance was evaluated using the same approach as in the thermal conductivity measurements described in section 5. Since the results (Figure 2 in the main document) do not show a significant difference between thin and thick sheets, the sheet-to-sheet contact resistance was deemed to be negligible and the effective resistivity was in turn assumed to be equal to the bulk resistivity.

At higher through–plane compression pressures, the thickness of the samples reduces, and the thickness value in Equation 16 should be therefore corrected to obtain reliable values. The relative increase in resistivity can be quantified as:

$$\frac{\rho_{th}}{\rho_{th,100kPa}} = \frac{\frac{aA_{th}}{(1-S)t_s}}{\frac{aA_{th}}{t_s}} = \frac{1}{1-S} = K_d, \tag{27}$$

where $\rho_{th,100kPa}$ is resistivity at the lowest reference pressure of 100 kPa, and S is the strain at the given pressure. In the last step in Equation 27, the term $\frac{1}{1-S}$ was identified to correspond to the density correction factor K_d, which was introduced in section 4. Figure SD8 shows that the maximum value of K_d is approximately 1.06 for the 0.55 gcm^{-3} sheet at 1080 kPa, which means that correcting the values of resistivity for the change in thickness would result in the maximum increase of 6 %. Since the relative uncertainty of resistivity for the 0.55 gcm^{-3} sheet at 100 kPa is 4 %, the correction was omitted as no significant improvement in the data quality can be achieved.

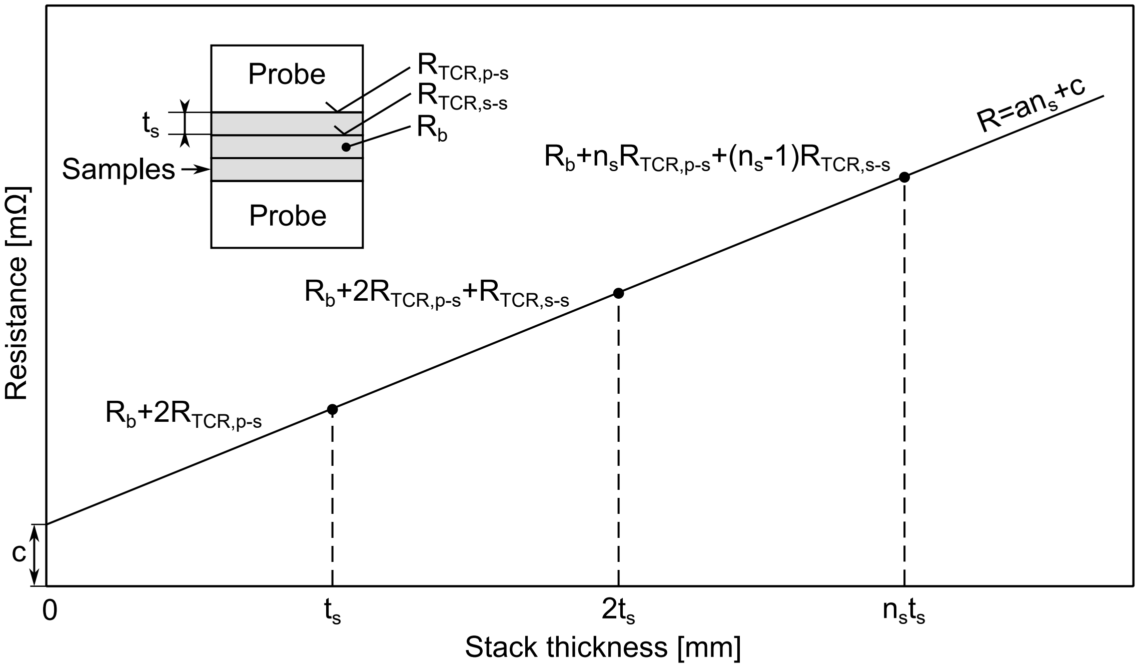

Figure SD15: Illustration of the resistance of a stack of sheets during the four-probe measurement.

7 Coefficient of thermal expansion

The measurements were performed using a TA Instruments TMA Q400 thermo-mechanical analyzer shown in Figure SD16a. Each of the measurement consisted of i) heating the sample to 100 °C at the rate of $4\,°\mathrm{Cmin}^{-1}$, ii) keeping the temperature constant for 10 min, iii) cooling the sample to 30 °C at the rate dictated by natural convection from the chamber, and repeating the i)-iii) steps at least three times. An example of the raw data is shown in Figure SD17. The probe compression force was set to 0.1 N, and nitrogen gas was introduced in the furnace at the flow rate of $50\,\mathrm{mLmin}^{-1}$. The cooling rate in the measurements performed later in the study was increased by flowing compressed air on the outside of the furnace. This allowed reducing the measurement time, which was previously limited by the low heat transfer rate during the cooling phase. The used instrument and measurement procedure were in agreement with ASTM E831-06. Stacking of the samples in the through–plane measurements deviated from the standard. An extended description of the measurement can be found in [SD4].

For the in–plane measurements, a total of 40 measurements was performed on a collection of samples randomly selected from 12 sample sets. Samples in each of the sample sets were cut from the calendered sheets described in section 1, and they were 10 mm wide and 15 to 29 mm long. The reported CTE values are an average of 306 heating cycles. The samples were mounted as shown in SD16b.

For the through–plane measurements, a total of 23 measurements was performed on a collection of samples randomly selected from 11 sample sets. The randomization included measuring single sheets and stacks to eliminate the possibility of high uncertainty at sample thicknesses lower than the 5 mm value recommendation by the device manufacturer. The reported CTE values are averages of 238 heating cycles. The samples were 10 mm squares and they were mounted as shown in SD16b. To confirm that stacking of samples does not affect the results, the through–plane CTE was plotted against the number of sheets in Figure SD18. For all the measurements, the difference between the results for varying number of sheets in the stack is within the measurement uncertainty, and the method is therefore considered valid.

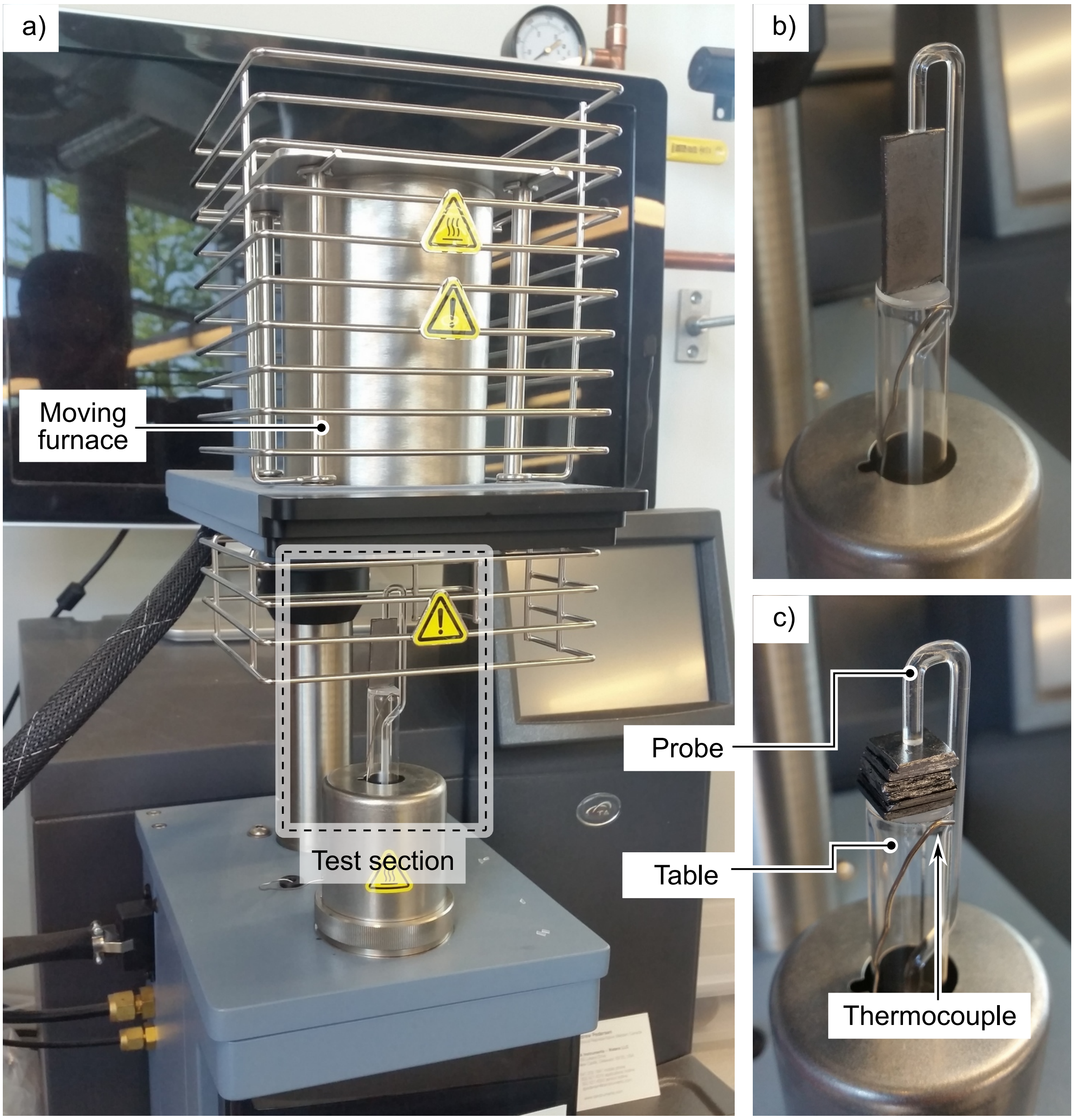

Figure SD16: a) A photograph of the TMA Q400 machine with the test section highlighted by the dashed line. In b) and c) the test section is showed with the sample for in–plane and through–plane measurements, respectively.

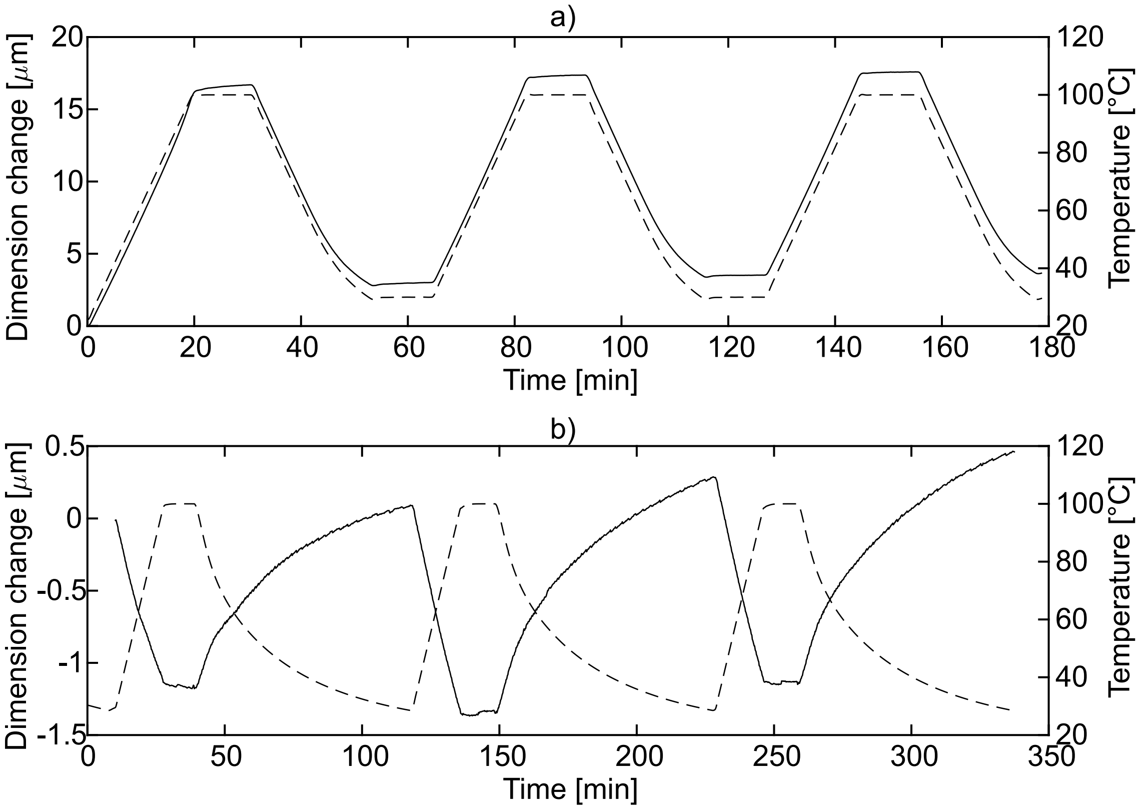

Figure SD17: A sample of the raw data for the $1.05\,\mathrm{gcm^{-3}}$ $140\,\mathrm{mgcm^{-2}}$ sample in a) through–plane and b) in–plane direction. In a) the cooling rate was increased by flowing compressed air around the furnace to reduce the measurement time. The solid lines represent the dimension change, and the dashed lines represent the temperature.

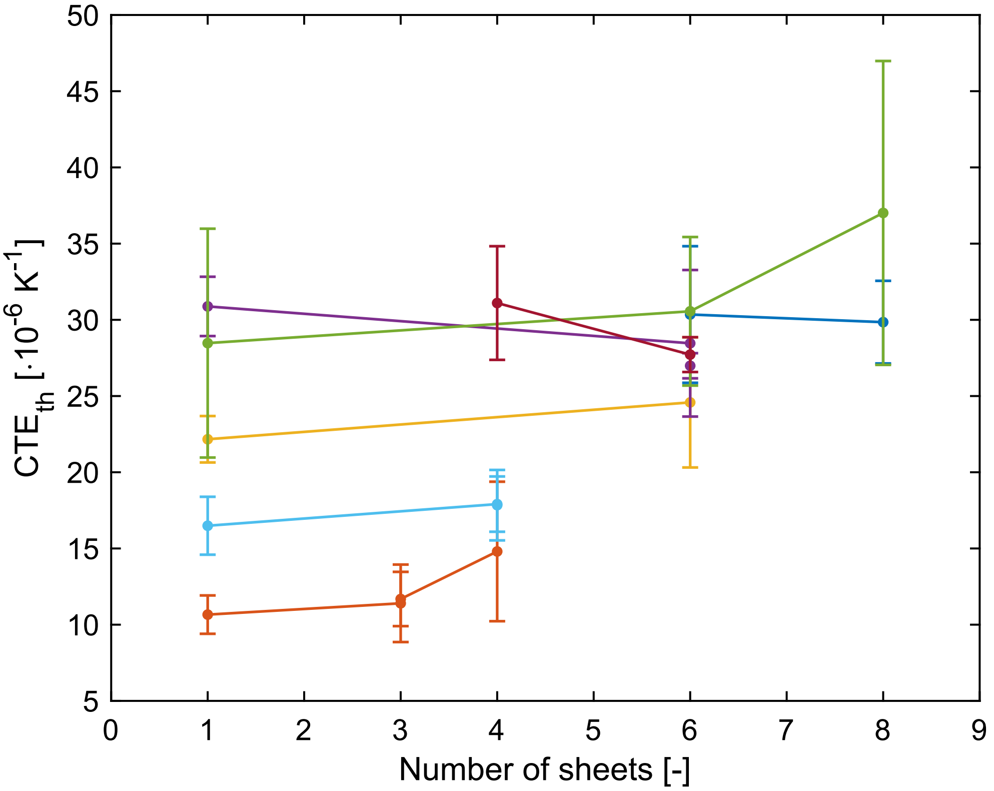

Figure SD18: The through–plane CTE as a function of number of sheets in the measured stack. Each of the colors represents one sample set.

To obtain the CTE, the raw data were plotted in the dimension-versus-temperature plot as shown in Figure SD19 and a line fit in the range 35 to 95 °C was determined for each of the cycles. The CTE values were calculated by dividing the slope of the line by the sample dimension. The final reported value of CTE is a mean value of all the cycles whose R^2 value is larger than 0.9. The criterion for the R^2 value was necessary to eliminate poor measurements arising from inevitable vibrations and other disturbances that occurred during the data collection period. The raw data files and the implementation of the data processing method is available in [SD3]. The data analysis method was chosen over the two-point one described ASTM E831-06 because it uses all collected data points and improves the robustness of the analysis.

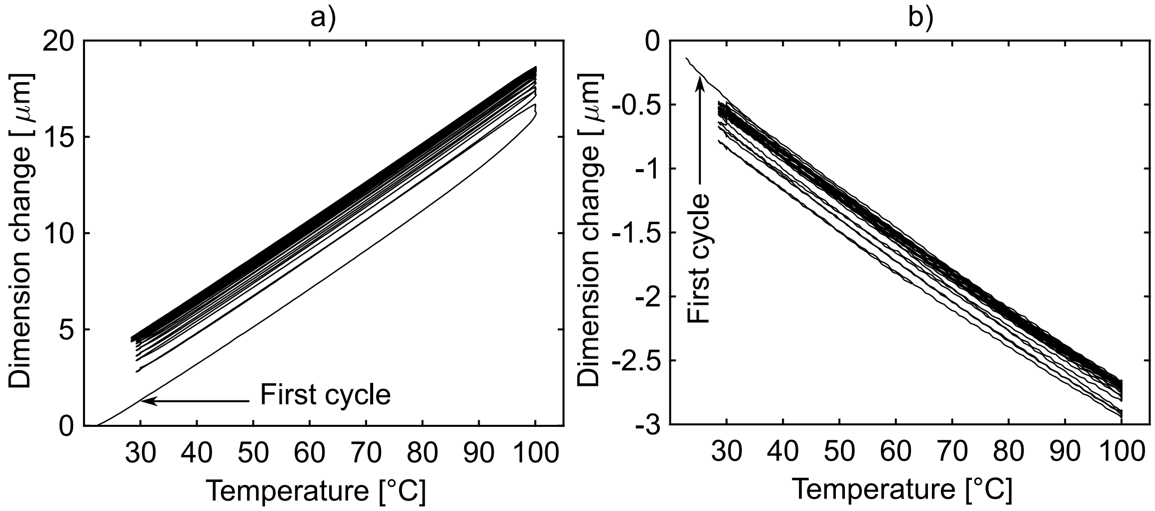

Figure SD19: A plot of dimension change as a function of temperature for the 1.05 gcm^{-3} 140 mgcm^{-2} sample in a) through–plane and b) in–plane direction.

8 Emissivity

Four 5 x 5 cm samples with the density of 0.55 g cm^{-3}, 1.05 g cm^{-3}, 1.54 g cm^{-3} and 1.7 g cm^{-3}, and the thickness of 2.76 mm, 1.43 mm, 0.96 mm and 0.84 mm, respectively, were cut from the 140 mgcm^{-2} calendered sheets that were described in section 1. The measurements were made using the Surface Optics Corporation 400T Fourier Transform Infrared Reflectometer [SD9] (Figure SD20) in accordance with the Method C of ASTM E408-13 Standard [SD2]. In the calibration process, the room was used as the black body, and a polished gold film as the reference for the spectral reflectivity (98 %). The samples were measured at room temperature and wavelengths ranging from 2 to 26 µm. Four measurements were made for each of the densities to capture the variation with the location and orientation of the sheet. The reflectometer outputs the near-normal-hemispherical spectral reflectivity at the accuracy of ±1 %. All the measurements were performed at 25 °C. Both the raw and processed data are available in [SD3].

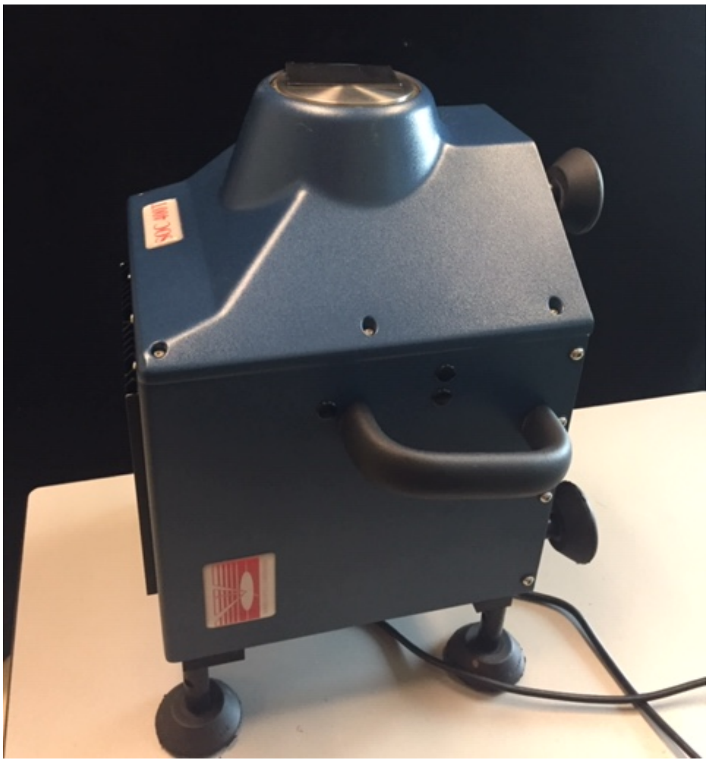

Figure SD20: A photograph of a Surface Optics Corporation 400T Fourier Transform Infrared Reflectometer that was used for measuring the emissivity.

9 Uncertainty analysis

9.1 Sample dimensions and area

The uncertainty of the thickness t of the measured sheets was determined as:

$$u_t = \sqrt{u_{t,d}^2 + \sigma_t}, \quad (28)$$

where $u_{t,d}$ is the micrometer accuracy of 0.0254 mm, and σ_m is the standard deviation of the thickness of six measured samples.

The uncertainty of the sample area A was calculated as

$$u_A = \frac{A_o - A_b}{2}, \quad (29)$$

where A_o is the area calculated based on the outer dimensions of the sample, and A_b is the area calculated based on the dimensions of the sample defined by the burr caused by cutting. For a circular sample, the burr and and the relevant dimensions are shown in Figure SD21. For this specific case, A_o and A_b are defined as:

$$A_o = \frac{\pi d_o^2}{4} \quad (30)$$

and

$$A_b = \frac{\pi d_b^2}{4}, \quad (31)$$

where d_o is the outer diameter, and d_b is the diameter defined by the size of the burr. The burr was considered to be the dominating source of uncertainty and the accuracy of the measuring instrument was therefore omitted.

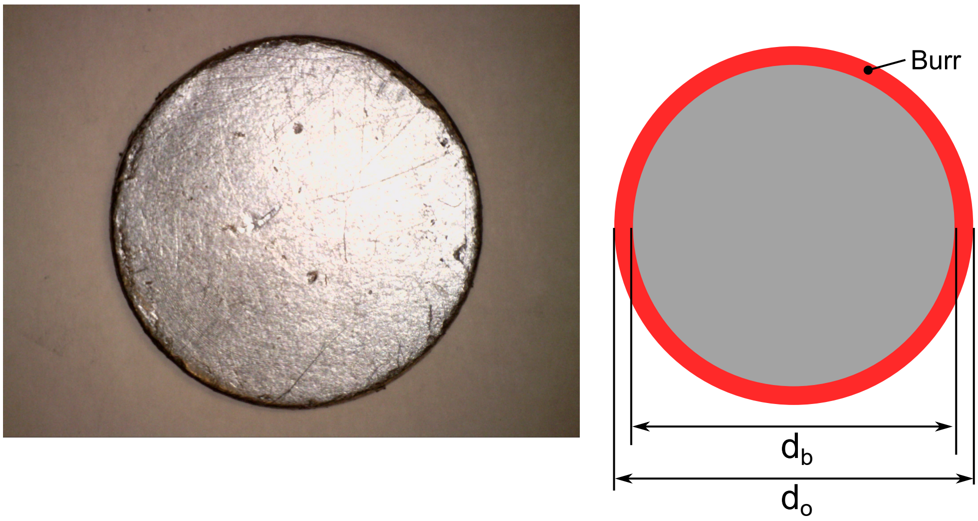

Figure SD21: Left: a photograph of one of the circular samples with visible burr on the perimeter. Right: a simplified illustration of the sample with the burr highlighted in red. The outer diameter d_o, and the diameter defined by the bur d_b are indicated.

9.2 Density

The density was calculated as:

$$d = \frac{m}{tA} = \frac{m}{V}, \tag{32}$$

where m, t, A, and V are the sample mass, thickness, surface area, and volume, respectively. The uncertainty was calculated using the partial derivative method as:

$$u_d = \sqrt{\left(\frac{\partial d}{\partial m}u_m\right)^2 + \left(\frac{\partial d}{\partial V}u_V\right)^2} = \sqrt{\left(\frac{1}{V}u_m\right)^2 + \left(\frac{-m}{V^2}u_V\right)^2}, \tag{33}$$

where u_m and u_V are the uncertainties of mass and volume, respectively. The uncertainty of mass was determined as:

$$u_m = \sqrt{u_{m,d}^2 + \sigma_m^2}, \tag{34}$$

where $u_{m,d}$ is the scale accuracy of 0.0002 g [SD12], and σ_m is the standard deviation of the mass of the six measured samples. The uncertainty of the sample volume was calculated as:

$$u_V = \sqrt{\left(\frac{\partial V}{\partial t}u_t\right)^2 + \left(\frac{\partial V}{\partial A}u_A\right)^2} = \sqrt{(Au_t)^2 + (tu_A)^2}, \tag{35}$$

where u_t is the uncertainty of the sample thickness and u_A is the uncertainty of the sample area

9.3 Compression pressure

Compression pressure was applied during the measurements of through–plane thermal and electrical conductivity. The uncertainty of pressure u_p was determined as:

$$u_p = \sqrt{\left(\frac{\partial p}{\partial F}u_F\right)^2 + \left(\frac{\partial p}{\partial A}u_A\right)^2} = \sqrt{\left(\frac{1}{A}u_F\right)^2 + \left(\frac{F}{A^2}u_A\right)^2}, \tag{36}$$

where p is the pressure, F is the force, and A is the sample area. The uncertainty of the force gauge reading is given as 0.1 % of the full scale [SD11]. However, due to the viscous behavior of NGS [SD5], the uncertainty of the force reading was expanded to $u_F = 10$ N.

9.4 Thermal conductivity and diffusivity

The uncertainty of the thermal conductivity u_k was calculated as:

$$u_k = \sqrt{u_{k,m}^2 + \sigma_k^2}, \tag{37}$$

where $u_{k,m}$ is the measurement accuracy of the TPS machine and σ_k is the standard deviation of the measurements. The value of $u_{k,m}$ reported by the manufacturer is 5 % of the reading [SD8]. The uncertainty of diffusivity u_α was calculated identically to the conductivity as:

$$u_\alpha = \sqrt{u_{\alpha,m}^2 + \sigma_\alpha^2}, \tag{38}$$

where $u_{\alpha,m}$ was set to be 5 % of the reading, identical to the conductivity. However, the equipment manual [SD8] does not specify the accuracy of the diffusivity. For a detailed analysis of the measurement uncertainty the implementation of the data processing within the Thermal Analyzer would have to be known. As this software is proprietary, an advanced uncertainty analysis was not performed.

9.5 Electrical conductivity

The propagation of the uncertainty through the data processing steps starts with the line fitting. The uncertainty of the line slope u_a was determined from the confidence interval of the slope, which was calculated using the *confint* function in Matlab. The confidence level was set to 68.27 %. The error of the resistance and voltage probe distance is not accounted for in the *confint* implementation.

The uncertainty of the resistivity u_ρ was calculated as:

$$u_\rho = \sqrt{\left(\frac{\partial \rho}{\partial a} u_a\right)^2 + \left(\frac{\partial \rho}{\partial A} u_A\right)^2} = \sqrt{(A u_a)^2 + (a u_A)^2} \tag{39}$$

The electrical conductivity is the inverse of resistivity and therefore the uncertainty of electrical conductivity u_σ is:

$$u_\sigma = \sqrt{(\frac{\partial \sigma}{\partial \rho} u_\rho)^2} = \sqrt{(-\rho^{-2} u_\rho)^2} = \rho^{-2} u_\rho. \tag{40}$$

The uncertainty of the pressure was calculated in an identical way as described in section 9.4.

9.6 Coefficient of thermal expansion

The uncertainty of CTE was calculated by grouping the values from all the cycles and calculating the standard deviation. This approach does not reflect the accuracy of the device. The manufacturer reported 0.1 % precision of CTE measurement and 15 nm resolution of the displacement measurements [SD16]. However, the information on the absolute accuracy of the length measurement was not provided [SD13], which limited the possibility to perform an advanced uncertainty analysis.

9.7 Emissivity

The uncertainty of the total emissivity u_ϵ was calculated as

$$u_\epsilon = \sqrt{u_{\epsilon,m}^2 + \sigma_\epsilon^2}, \tag{41}$$

where $u_{\epsilon,m}$ is the measurement accuracy of the device (1 %) and σ_ϵ is the standard deviation of the four measurement repetitions. The 1 % measurement accuracy $u_{\epsilon,m}$ does not account for the propagation of the uncertainty through the spectral averaging process. The ASTM standards do not provide a clear guideline on how to address the propagation. It is likely that the error is amplified during the spectral averaging process, however, addressing this issue was out of the scope of this work.

References

[SD1] Hot disk thermal constants analyser - instruction manual - revision date 2016-11-08, 2016.

[SD2] ASTM. Standard Test Methods for Total Normal Emittance of Surfaces Using Inspection-Meter, E408 - 13. *ASTM International*, 2013.

[SD3] M. Cermak, N. Perez, M. Collins, and M. Bahrami. Material properties and structure of natural graphite sheet - dataset. https://doi.org/10.20383/101.0216, 2020.

[SD4] Martin Cermak. *Natural graphite sheet heat sinks for power electronics.* PhD thesis, Simon Fraser University, 2020. In preparation.

[SD5] Martin Cermak and Majid Bahrami. Compression behavior of natural graphite sheet. *SN Applied Sciences*, 2(3):357, 2020.

[SD6] Mattias Gustavsson, Ernest Karawacki, and Silas E. Gustafsson. Thermal conductivity, thermal diffusivity, and specific heat of thin samples from transient measurements with hot disk sensors. *Review of Scientific Instruments*, 65(12):3856–3859, 1994.

[SD7] Yi He. Rapid thermal conductivity measurement with a hot disk sensor: Part 1. theoretical considerations. *Thermochimica Acta*, 436(1):122 – 129, 2005.

[SD8] Hot Disk AB. TPS 2500 S Hot Disk Thermal Constants Analyser. `https://www.hotdiskinstruments.com/content/uploads/2017/03/2500S.pdf`. Online; accessed 13 June 2020.

[SD9] Donald A. Jaworske and Timothy J. Skowronski. Portable infrared reflectometer for evaluating emittance. *AIP Conference Proceedings*, 504(1):791–796, 2000.

[SD10] B. T. (Brian Thomas) Kelly. *Physics of graphite / B.T. Kelly.* Applied Science, London ; Englewood, N.J., 1981.

[SD11] Mark-10 Corporation. Advanced Digital Force Gauges Series 5 - Data sheet 32-1111 REV 0119. `http://www.mark-10.com/pdf/DataSheetSeries5.pdf`. Online; accessed 13 June 2020.

[SD12] Ohaus Corporation. Adventurer Analytical and Precision Balances - 80774742 I 20181105. `https://dmx.ohaus.com/WorkArea/showcontent.aspx?id=4294968488`. Online; accessed 13 June 2020.

[SD13] Yue Shuman. email communication. Applications Support Engineer at TA Instruments.

[SD14] Martin Smalc, Julian Norley, R. Andy Reynolds, Richard Pachuta, and Dan W Krassowski. Advanced Thermal Interface Materials Using Natural Graphite. In *2003 International Electronic Packaging Technical Conference and Exhibition, Volume 2*, pages 253–261, 2003.

[SD15] H.M. Spencer. Empirical heat capacity equations of gases and graphite. *Ind. and Eng. Chem.*, Vol: 40, 11 1948.

[SD16] TA Instruments. TMA Q400EM/Q400 Brochure L90033.001. `https://www.tainstruments.com/wp-content/uploads/TMA.pdf`. Online; accessed 13 June 2020.

[SD17] L.W. Wang, S.J. Metcalf, R.E. Critoph, R. Thorpe, and Z. Tamainot-Telto. Thermal conductivity and permeability of consolidated expanded natural graphite treated with sulphuric acid. *Carbon*, 49(14):4812 – 4819, 2011.
